# Recent Breakthroughs in Cyclizations of *Ortho*-Quinone Methides

**DOI:** 10.3390/molecules31111846

**Published:** 2026-05-27

**Authors:** Dongyi Wang, Jiahao Guo, Linzhi Tan, Liqin Qiu, Chitreddy V. Subba Reddy, Wang Xia

**Affiliations:** 1 School of Environmental and Chemical Engineering, Wuyi University, Jiangmen 529020, China; 2112423012@wyu.edu.cn (D.W.); 2112523037@wyu.edu.cn (J.G.); 2112523042@wyu.edu.cn (L.T.); 2 School of Chemistry, IGCME, Guangdong Key Lab of Chiral Molecules and Drug Discovery, Sun Yat-Sen University, Guangzhou 510006, China; 3 Chemistry Services, Aragen Lifesciences Pvt. Ltd., Survey Nos: 125 (Part) & 126, IDA Mallapur, Hyderabad 500076, Telangana, India

**Keywords:** *Ortho*-quinone methides, organic synthesis, [4 + n] cyclization, asymmetric catalysis, heterocyclic synthesis

## Abstract

*Ortho*-quinone methides (*o*-QMs) constitute a class of highly reactive and versatile intermediates in organic synthesis, characterized by a unique polarized electronic structure that combines an electron-deficient quinoid ring with an exocyclic electrophilic methylene or alkylidene unit. This distinctive feature renders powerful C4 synthons capable of participating in a wide range of higher-order cyclization reactions. In particular, [4 + n] cyclizations involving *o*-QMs (e.g., [4 + 1], [4 + 2], and [4 + 3]) have emerged as effective strategies for the rapid construction of structurally complex carbocyclic and heterocyclic frameworks, many of which serve as privileged scaffolds in natural products and functional materials. This review provides a comprehensive overview of recent advances in this rapidly developing field, with a systematic discussion of reaction design, mechanistic insights, and synthetic applications across various [4 + n] cyclization modes. Furthermore, current challenges are critically evaluated, and future opportunities are proposed, particularly in the development of novel catalytic systems, asymmetric variants, and innovative *o*-QM precursors. Overall, this review aims to offer researchers a clear understanding of the state of the art in *o*-QM chemistry and to inspire further innovation in this dynamic area of organic synthesis.

## 1. Introduction

Oxygen-containing heterocycles represent key structural motifs in natural products, pharmaceuticals, and functional materials [1,2,3]. The efficient construction of such complex cyclic architectures remains a central objective and driving force in modern organic chemistry. In this context, cyclization reactions, particularly higher-order variants, are especially valuable due to their ability to rapidly generate molecular complexity in a single, atom-economical step. Among the diverse synthons employed, *ortho*-quinone methides (*o*-QMs) and their derivatives have emerged as versatile C4 building blocks in [4 + n] cyclization reactions, owing to their distinctive polarized electronic structure. This strategy provides a powerful and modular platform for the construction of five- to seven-membered ring systems [4,5,6].

**Scheme 1:**
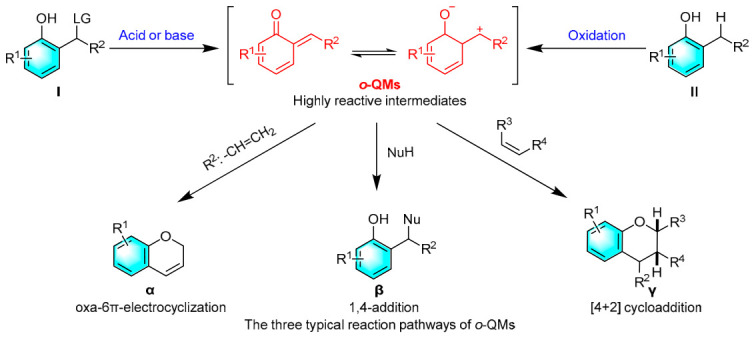
Generation of *o*-QMs and synthetic application.

As highly reactive intermediates, *o*-QMs have found widespread applications in organic synthesis, natural product synthesis, materials chemistry, and the development of bioactive compounds [7,8,9,10,11,12,13]. Structurally, they consist of an electron-deficient quinoid ring conjugated with a highly electrophilic exocyclic methylene unit. Typically, *o*-QMs participate in three primary types of reactions: (1) 1,4-addition with nucleophiles, (2) [4 + 2] cycloaddition with dienophiles, and (3) oxa-6π electrocyclization. Cyclization reactions are defined as chemical transformations that construct cyclic structures, including aromatic and non-aromatic heterocycles and carbocycles, which are utilized in various fields such as pharmaceuticals and crop protection [14]. A prominent subclass is cycloadditions, which are classified as concerted (synchronous or asynchronous) or stepwise, the latter proceeding via zwitterionic or biradical intermediates. The buta-1,3-diene–ethylene reaction exemplifies a synchronous concerted pathway. Asynchronous concerted processes involve nonsimultaneous σ-bond formation. Zwitterionic intermediates arise only under highly polarized conditions, defining the stepwise zwitterionic regime [4]. However, *o*-QMs’ high reactivity and inherent instability often preclude their isolation, and thus most synthetic applications rely on in situ generation. Common strategies for generating *o*-QMs include pyrolysis, photolysis, tautomerization, and acid- or base-promoted elimination of suitable precursors. Among these, acid/base-promoted methods and biomimetic oxidation of *o*-alkylphenols are the most widely employed. Nevertheless, the latter often requires toxic heavy-metal oxidants, which may limit practical applicability (Scheme 1). The remarkable reactivity and amphoteric nature of *o*-QMs arise from their conjugated exocyclic double bond and aromatic system, forming a delocalized 4π-electron framework. This unique electronic structure enables *o*-QMs to function as versatile 4π components in diverse cycloaddition processes, making them powerful platforms for constructing complex carbocyclic and heterocyclic architectures. Building on this fundamental 4π-electron donor characteristic, research over the past decade has expanded significantly beyond classical [4 + 2] cycloadditions. Systematic investigations of their reactivity with various π-systems have led to the development of a broad range of well-controlled [4 + n] cyclization strategies.

In this review, we have highlighted recent advances in *o*-QM cycloaddition chemistry over the past decade, with particular emphasis on innovations in cyclization strategies. First, [4 + 1] cyclizations for the construction of five-membered rings were discussed, focusing on efficient coupling with C1 synthons. Next, advances in [4 + 2] cycloadditions, especially in asymmetric catalysis and green synthesis, were summarized. Finally, emerging [4 + 3] and other unconventional cyclization modes were analyzed, showcasing their unique potential in constructing complex cyclic frameworks. Through this systematic overview, we aim to illustrate the evolution of the field from empirical discovery to rational design, and to provide perspectives for the future development in synthetic chemistry.

## 2. [4 + 1] Cyclization Reactions: Access to Five-Membered Heterocycles

[4 + 1] Cyclization represents a powerful and convergent strategy for the construction of five-membered heterocycles, particularly the biologically significant 2,3-dihydrobenzofuran scaffold. This motif often features a C2 all-carbon quaternary center, a structural element frequently encountered in bioactive natural products and pharmaceuticals. Despite its synthetic value, the development of [4 + 1] cyclization reactions have long been hindered by two key challenges: (1) the intrinsically high activation energy associated with the cycloaddition process, and (2) the propensity of reactive carbene or carbene-like intermediates to undergo competitive side reactions, most notably cyclopropanation, which may limit the general applicability of these transformations.

### 2.1. Transition-Metal-Free [4 + 1] Cyclization

**Scheme 2:**
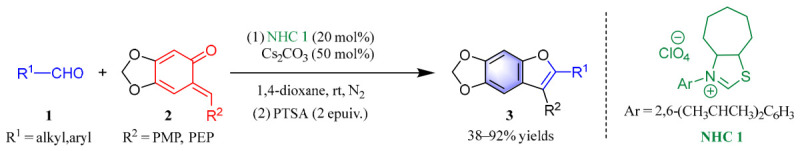
NHC-catalyzed tandem annulation of *o*-QMs with aldehydes.

Yao (2016) reported an **NHC 1**-catalyzed [4 + 1] cyclization of aldehydes **1** with *o*-QMs **2** to form 2,3-disubstituted benzofurans **3** (Scheme 2) [15]. From the established typology, this cascade strategy avoids metal residues and operates at room temperature (compared to metal-catalyzed cyclizations), and features good atom economy (with water as the only byproduct overall). However, its efficiency is highly substituent-dependent: electron-withdrawing aryl aldehydes give yields of 70–90%, whereas electron-donating aryl aldehydes give only 53–62%. Only one alkyl aldehyde (*n*-butyraldehyde, 70%) was demonstrated, and an additional acid catalyst (*p*-toluenesulfonic acid) is required for the second cyclization step. Therefore, this method is suitable for electron-deficient aryl aldehydes; for electron-rich or aliphatic substrates, metal-catalyzed cyclization may still be a more reliable choice.

**Scheme 3:**
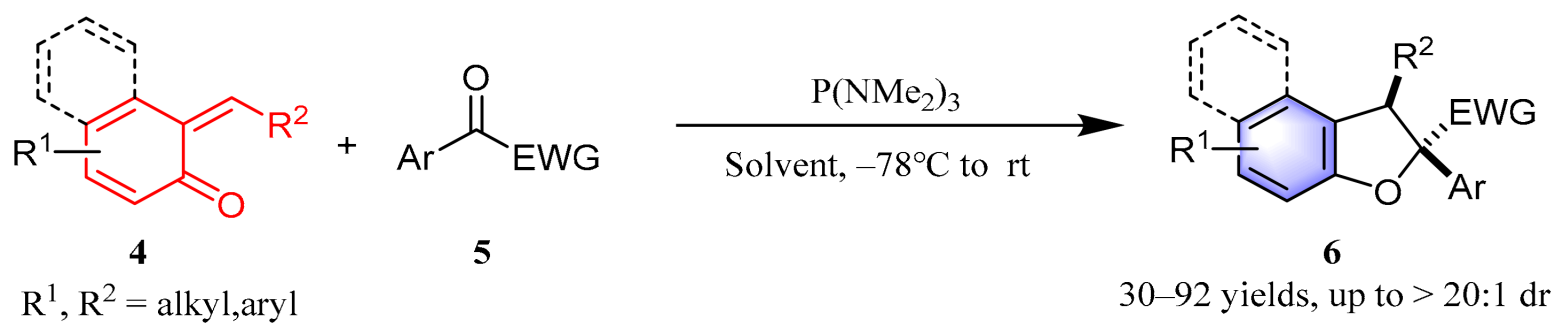
Phosphorus(III)-mediated formal [4 + 1]-cycloaddition between 1,2-dicarbonyls and *o*-QMs.

Ashfeld (2016) reported a P(III)-mediated formal [4 + 1] cycloaddition of *o*-QMs **4** with 1,2-dicarbonyls compounds **5** to form 2,3-dihydrobenzofurans **6** with a C2 quaternary center (Scheme 3) [16]. From the established typology, this is a pure cycloaddition. Atom economy is moderate due to stoichiometric phosphine oxide waste. Mechanistically, the reaction proceeds via a dioxyphospholene/zwitterion equilibrium, with P=O bond formation as the thermodynamic driving force. However, the method excels in stereoselectivity (up to ≥20:1, with E/Z convergence to syn-product). Limitations include poor tolerance of alkyl/vinyl β-substituents and better performance with electron-deficient arenes. Thus, from a cycloaddition perspective, this method is preferred when stereocontrol is critical.

**Scheme 4:**
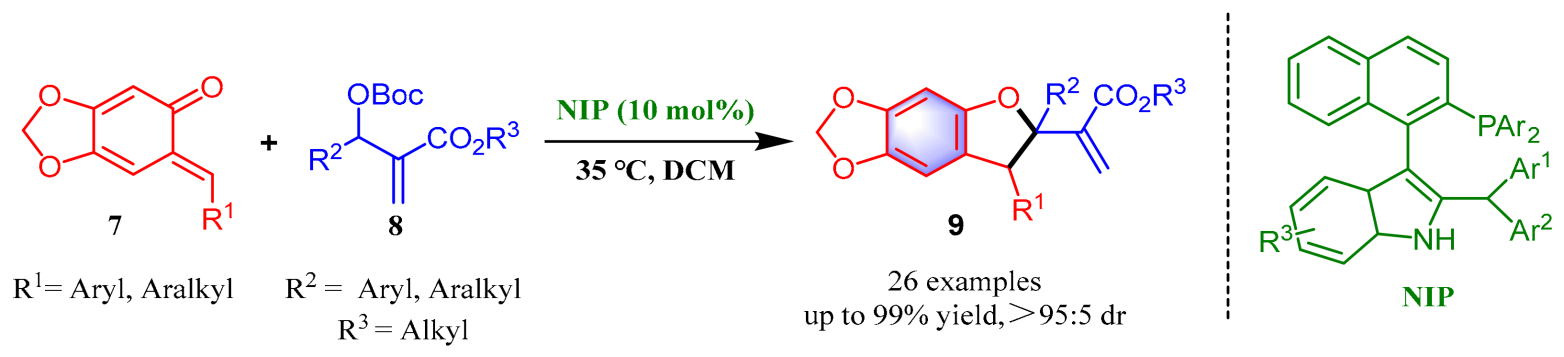
NIP-catalyzed [4 + 1] cyclization of *o*-QMs with MBH carbonates.

Shi (2018) reported the first **NIP**-catalyzed [4 + 1] cyclization of *o*-QMs **7** with MBH carbonates **8** (Scheme 4) [17]. From the established typology, this phosphine-mediated cycloaddition uses the MBH carbonate as a C1 synthon via an allylic phosphorus ylide. Atom economy is good (catalytic phosphine, CO_2_ byproduct). The method affords dihydrobenzofurans **9** in up to 99% yield and >95:5 d.r. with broad substrate scope. Mechanistically, the reaction proceeds via an allylic phosphorus ylide intermediate. However, a limitation is the low enantioselectivity (24–26% ee) achieved with chiral **NIPs**. Thus, from a cycloaddition perspective, this method excels in diastereocontrol but is not yet suitable for asymmetric synthesis.

**Scheme 5:**
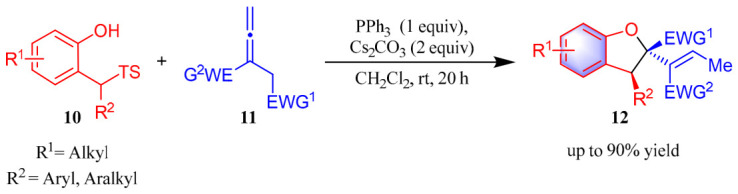
PPh_3_-catalyzed [4 + 1] cyclization of dienate esters with *o*-QMs.

Also in 2018, Waser disclosed a PPh_3_-catalyzed [4 + 1] cyclization of *o*-QMs with α-branched allenoates **11** (Scheme 5) [18]. From the established typology, the allenoate functions as a C1 synthon via its β′-position—an unusual reactivity mode. Mechanistically, the reaction proceeds via phosphine addition, proton transfer, and 5-exo-trig cyclization, with deuterium labeling confirming multiple proton transfers. Atom economy is moderate due to stoichiometric PPh_3_ (1 equiv) and Cs_2_CO_3_ (2 equiv). Limitations include narrow allenoate scope (substituted allenoates give only 30–40% yields) and, for *ortho*-substituted *o*-QMs, minor [4 + 3] byproducts. Diastereoselectivity is excellent, but enantioselective variants failed. Thus, from a cyclization perspective, this work introduces a novel C1 reactivity mode for allenoates, but the stoichiometric phosphine loading and narrow scope limit its synthetic utility.

**Scheme 6:**
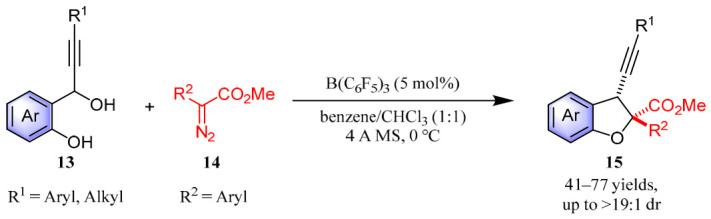
B(C_6_F_5_)_3_-catalyzed formal [4 + 1] cycloaddition of between alkyne-tethered *o*-QMs and diazoacetates.

Sun group (2019) reported a B(C_6_F_5_)_3_-catalyzed formal [4 + 1] cycloaddition of alkyne-tethered *o*-QMs with diazoacetates **14** (Scheme 6) [19]. From the established typology, this Lewis acid-catalyzed cycloaddition uses the diazoacetate as a C1 synthon via a metal-free carbene intermediate. Atom economy is good (catalytic B(C_6_F_5_)_3_, N_2_ byproduct). The method affords dihydrobenzofurans **15** with a quaternary carbon center in good yields (up to 77%) and high diastereoselectivities (>19:1 d.r.). However, limitations include moderate substrate scope and the need for low temperature (0 °C), mixed solvents, and molecular sieves. Thus, from a cycloaddition perspective, this method offers a metal-free, highly diastereoselective route to quaternary dihydrobenzofurans, but the specific conditions limit its practicality.

**Scheme 7:**
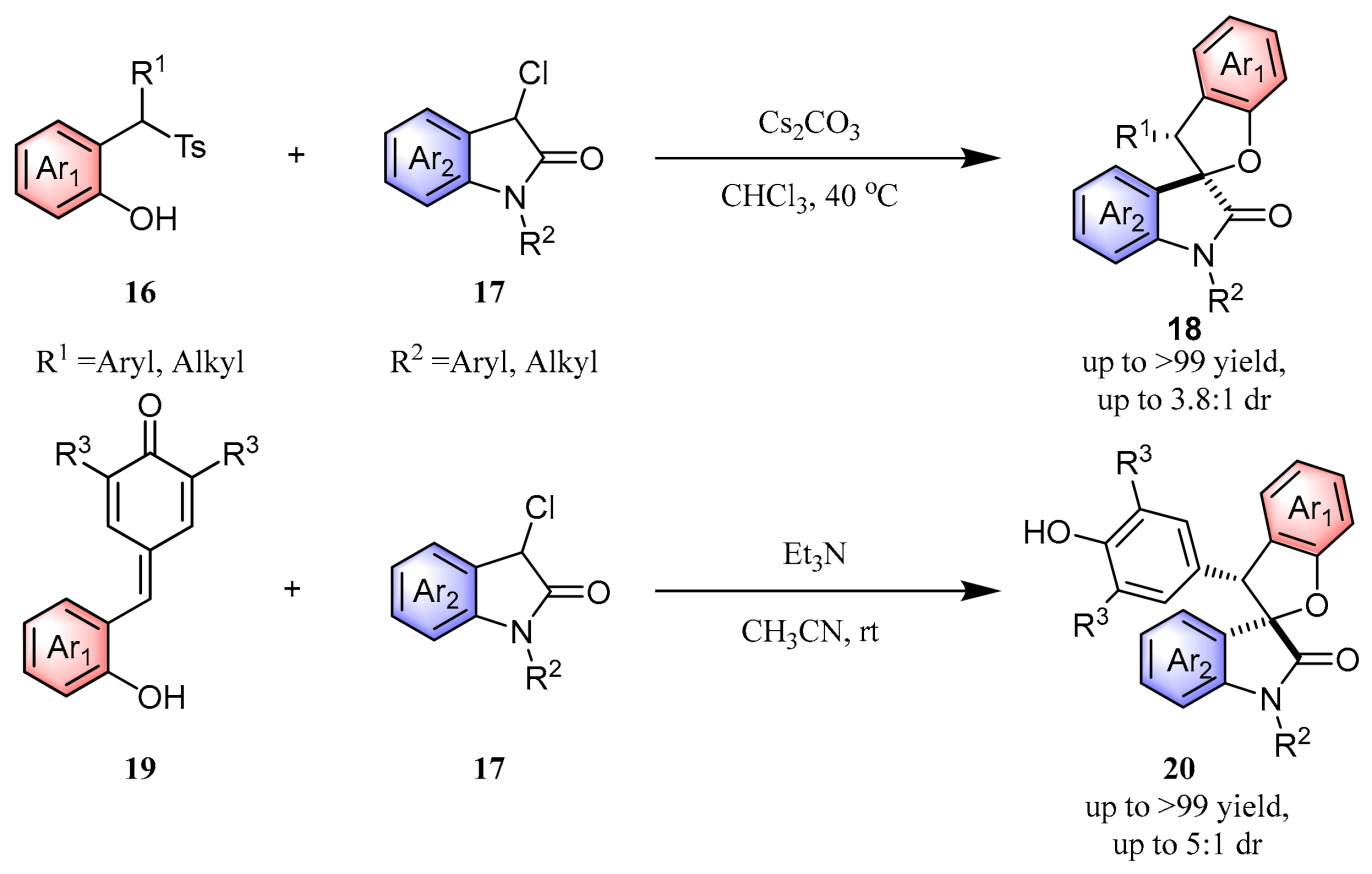
Base-promoted formal [4 + 1] cycloaddition of *ortho*- or *para*-quinone methides with 3-chlorooxindoles.

Wang and co-workers reported a base-promoted formal [4 + 1] cycloaddition of *o*-/*p*-QMs **16**/**19** with 3-chlorooxindoles **17** in 2021 (Scheme 7) [20]. From the established typology, this base-mediated cycloaddition uses 3-chlorooxindole as a C1 synthon via a carbanionic intermediate. Atom economy is good (metal-free). The method affords spirooxindoles **18** in high yields (up to >99%) under mild conditions. Mechanistically, computational studies revealed that 1,4- vs. 1,6-addition configurations lead to opposite diastereoselectivity. The diastereoselectivity varies depending on the substrate (dr ranging from 1:1 to >19:1, with typical values between 1.4:1 and 5:1), and the reaction requires 2.5 equiv of Cs_2_CO_3_. Thus, from a cycloaddition perspective, this method provides a metal-free, high-yielding route to spirooxindoles with tunable diastereoselectivity depending on the QM type.

**Scheme 8:**
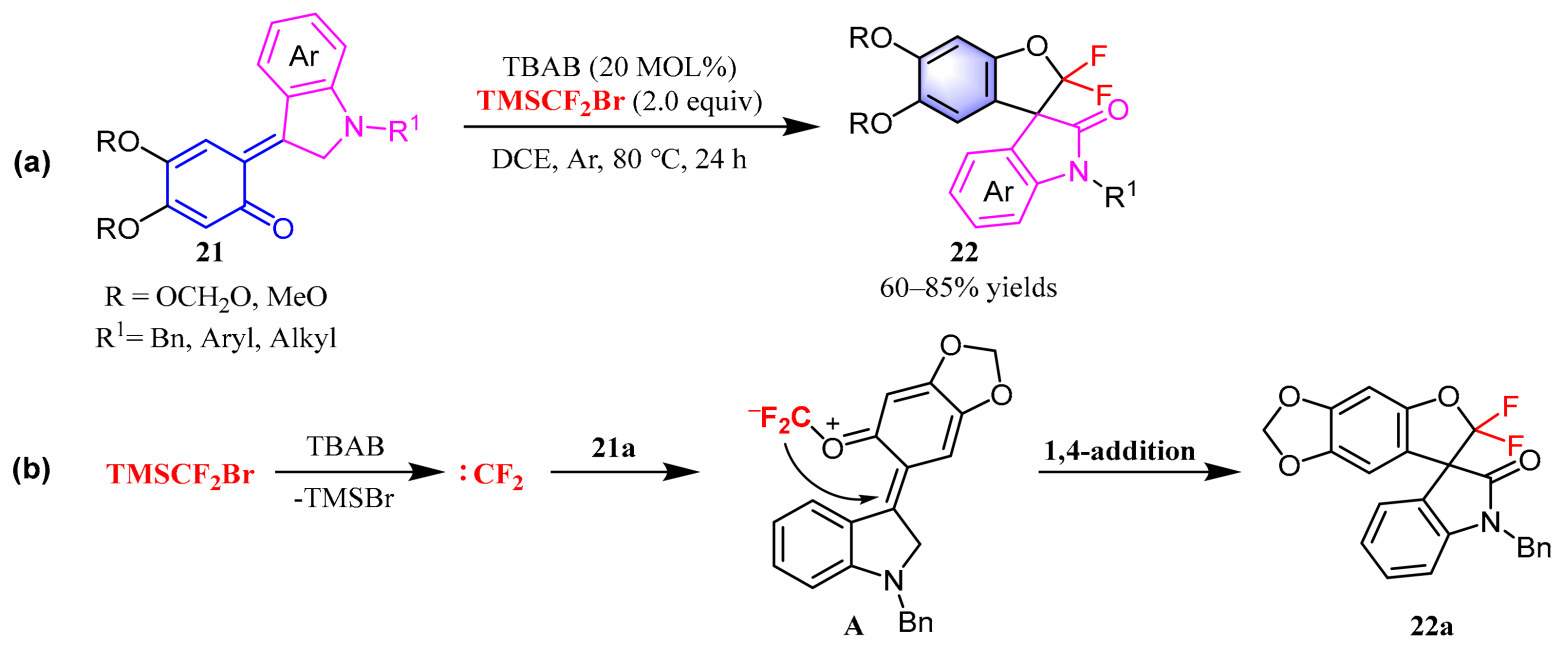
(**a**) Metal-free formal [4 + 1] cycloaddition of difluorocarbene and (**b**) the possible mechanism.

In the same year, Yang, Jiang, and co-workers reported a metal-free formal [4 + 1] cycloaddition of difluorocarbene with *o*-QMs **21** (Scheme 8a) [21]. From the established typology, this cycloaddition uses TMSCF_2_Br as the difluorocarbene precursor, serving as a C1 synthon. Atom economy is good (metal-free, base-mediated). The method affords gem-difluorinated 2,3-dihydrobenzofurans **22** in yields ranging from 60% to 85% with broad substrate scope, tolerating various electron-donating and electron-withdrawing substituents. Mechanistically(Scheme 8b), control experiments with radical scavengers ruled out radical pathways, supporting a difluorocarbene-mediated process. The reaction proceeds via initial trapping of difluorocarbene by the oxygen atom of *o*-QM, followed by intramolecular 1,4-nucleophilic attack. Thus, from a cycloaddition perspective, this method provides a metal-free entry to difluorinated dihydrobenzofurans with high efficiency and regiocontrol.

**Scheme 9:**
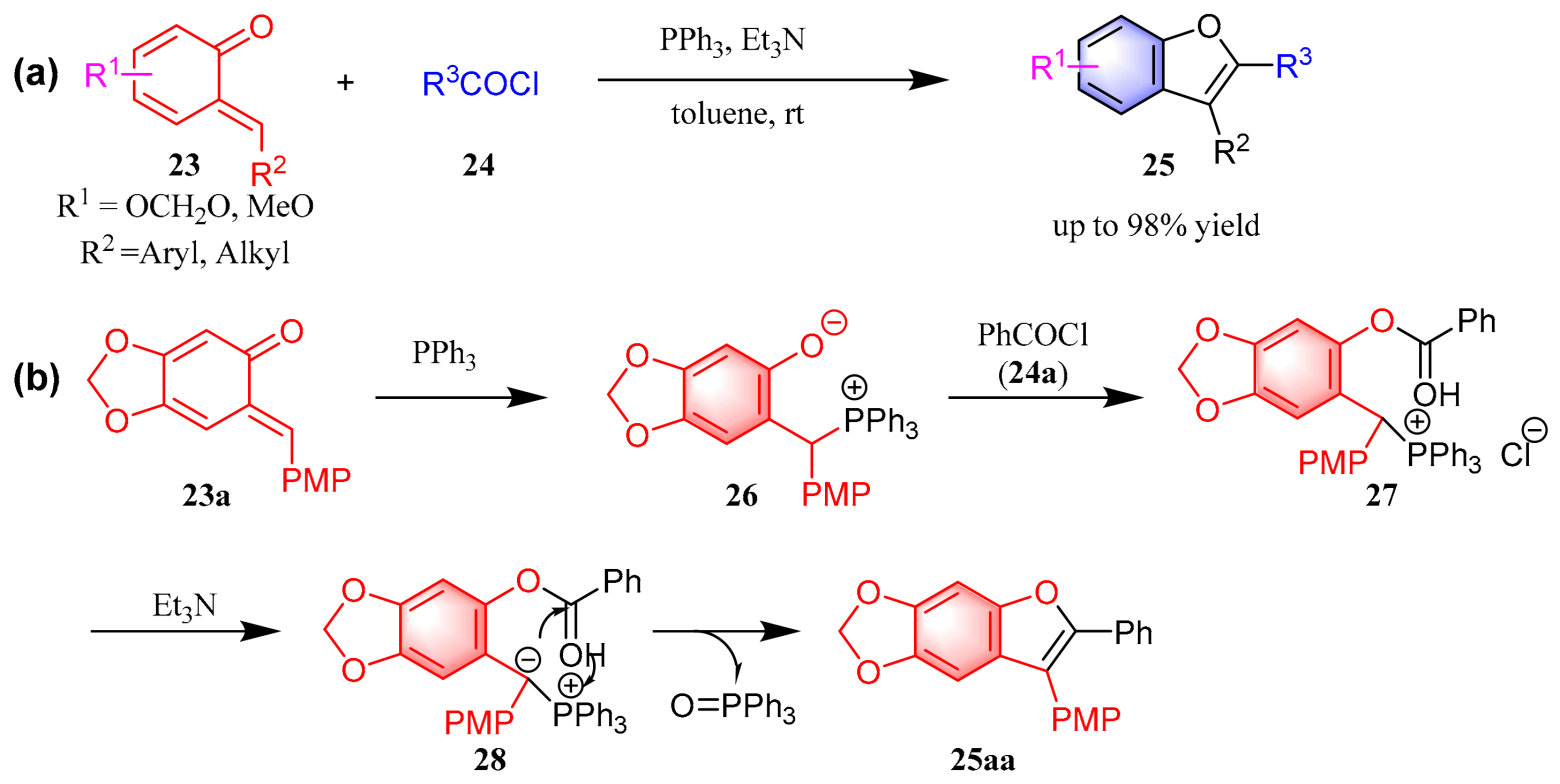
(**a**) PPh_3_-triggered [4 + 1] cyclization of *o*-QMs with acyl chlorides and (**b**) the possible mechanism.

In 2022, Yang, Wang, and co-workers described a PPh_3_-triggered tandem reaction between *o*-QMs **23** and acyl chlorides **24** (Scheme 9a) [22]. From the established typology, this [4 + 1] cyclization proceeds via phospha-Michael addition, *O*-acylation, and intramolecular Wittig reaction, affording benzofurans **25** in good to excellent yields (up to 98%). This work represents the first example of employing non-carbon nucleophiles in [4 + 1] cyclizations of *o*-QMs. Mechanistically (Scheme 9b), PPh_3_ adds to *o*-QM **23a** (rearomatization driven) to give phosphonium intermediate **26**, which undergoes *O*-acylation with **24a** to form **27**. Deprotonation with Et_3_N generates ylide **28**, and subsequent intramolecular Wittig reaction releases Ph_3_PO and delivers **25aa**. The reaction generates triphenylphosphine oxide as a stoichiometric byproduct. Thus, from a cyclization perspective, this method provides conceptual novelty through non-carbon nucleophile activation, with the generation of stoichiometric phosphine oxide as an inherent feature of the Wittig step.

**Scheme 10:**
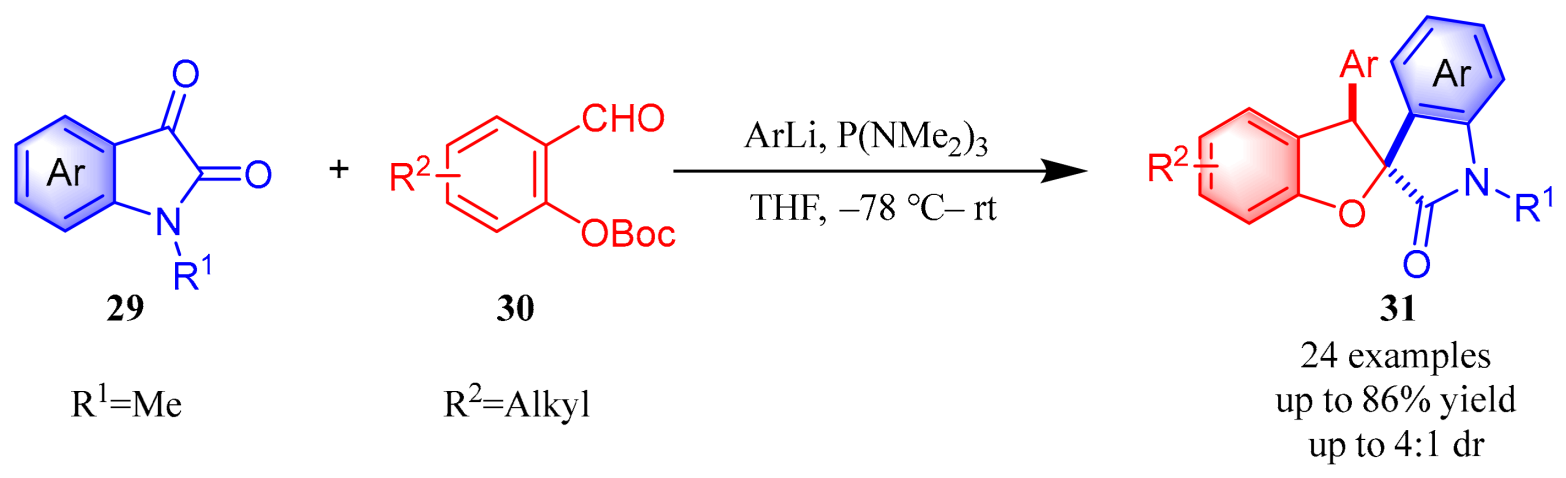
Phosphorus-mediated formal [4 + 1] cycloaddition of *o*-QMs with isatin-derived oxyphosphonium enolates.

Ashfeld’s group further reported a phosphorus-mediated formal [4 + 1] cycloaddition for the synthesis of spirooxindole-fused benzofurans **31** using isatin-derived oxyphosphonium enolates (Scheme 10) [23]. From the established typology, this transformation represents a formal [4 + 1] cycloaddition. The reaction employs P(NMe_2_)_3_-activated isatins **29** and in situ-generated *o*-QMs **30** from salicylaldehydes, affording spirocycles in yields up to 86%, with diastereoselectivities up to 4:1 dr. Atom economy is moderate due to stoichiometric phosphorus reagent. Mechanistic studies (variable-temperature NMR) revealed a dynamic equilibrium between cyclic and acyclic intermediates, governing reactivity and selectivity. Sterically demanding aryllithium suppressed benzopyran byproduct formation. Thus, from a cycloaddition perspective, this work provides a metal-free route to spirooxindoles and insights into Kukhtin–Ramirez equilibria.

**Scheme 11:**
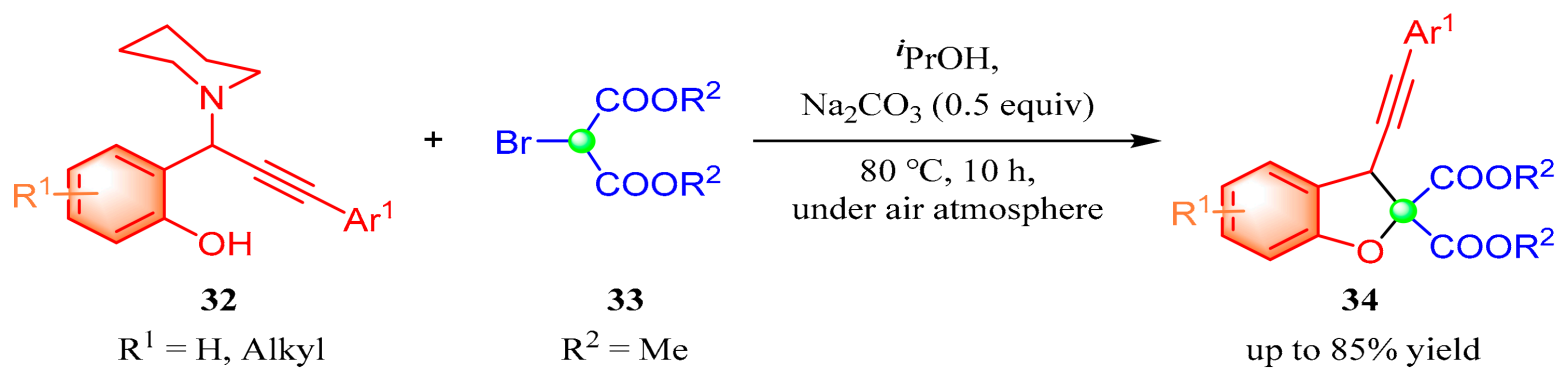
[4 + 1] cyclization reactions involving alkynyl *o*-QMs.

In 2025, Kong and co-workers developed a base-mediated [4 + 1] cyclization of alkynyl *o*-QM precursors **32** with 2-bromomalonates **33** (Scheme 11) [24]. From the established typology, this transformation proceeds via conjugate addition/intramolecular substitution. The reaction operates under air in undried isopropanol, affording functionalized dihydrobenzofurans **34** in up to 85% yield. Atom economy is moderate due to stoichiometric base and HBr byproduct. Mechanistic studies confirmed the essential role of the *ortho*-hydroxyl group for in situ *o*-QM generation. Thus, from a cyclization perspective, this protocol offers a practical route to dihydrobenzofurans under simple conditions.

### 2.2. Transition-Metal-Catalyzed [4 + 1] Cyclization

**Scheme 12:**
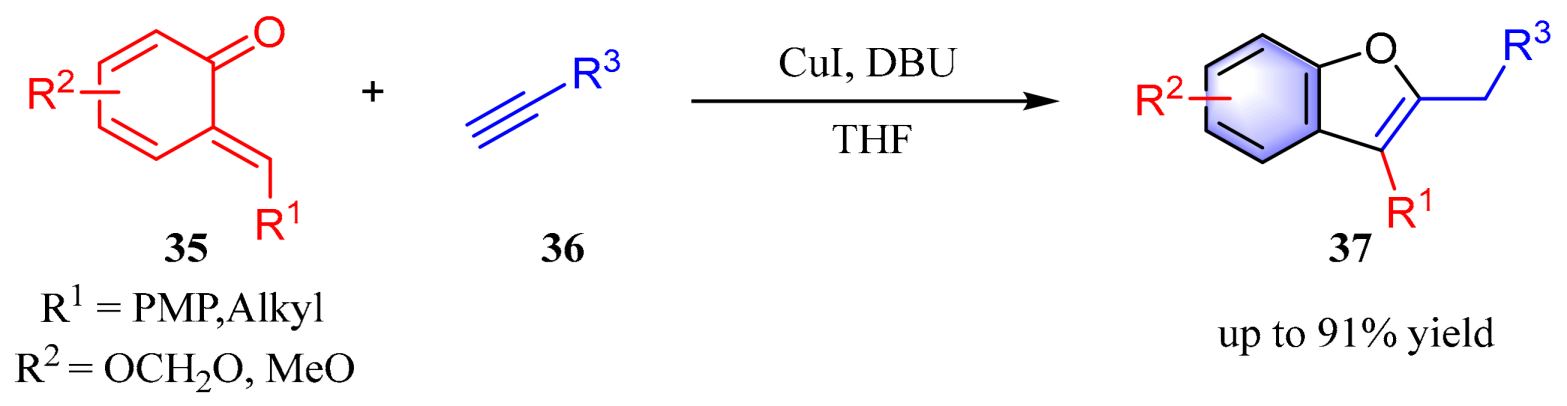
Cu(I)-catalyzed formal [4 + 1] cycloaddition of *o*-QMs with terminal alkynes.

Recently in 2025, Wang and co-workers reported a Cu(I)-catalyzed formal [4 + 1] cycloaddition between *o*-QMs **35** and terminal alkynes **36** (Scheme 12) [25]. From the established typology, this transformation affords 2,3-disubstituted benzofurans **37** in good to high yields (44 examples, up to 91%) under mild conditions with catalytic CuI (2 mol%) and DBU (0.05 equiv). Atom economy is good due to catalytic amounts of metal and base. The method tolerates a wide range of terminal alkynes and works with both stable and in situ-generated *o*-QMs, including a one-pot tandem process. However, certain *o*-QMs bearing styryl or vinyl substituents led to side reactions (6π-electrocyclization or decomposition), limiting the substrate scope. Thus, from a cycloaddition perspective, this protocol provides a practical and atom-economical entry to benzofurans, albeit with some restrictions on the *o*-QM structure.

### 2.3. Asymmetric Catalysis in [4 + 1] Cyclization

**Scheme 13:**
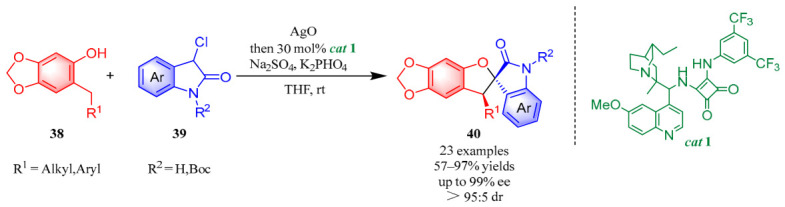
Squaramide-catalyzed asymmetric [4 + 1] cyclization of *o*-QMs with 3-chlorooxindoles.

In 2017, Jiang, Shi and co-workers developed a catalytic asymmetric [4 + 1] cyclization of *o*-QMs **38** with 3-chlorooxindoles **39** using chiral squaramide **cat 1**, affording spirooxindole-based dihydrobenzofurans **40** in up to 97% yield, >95:5 dr, and 99% ee (Scheme 13) [26]. A one-pot domino oxidation version from 2-alkylphenols **38** was also realized, offering step economy. Both protocols tolerate broad substrates and were scalable. Thus, from a cyclization perspective, this work represents the first highly enantioselective [4 + 1] cyclization of *o*-QMs.

**Scheme 14:**
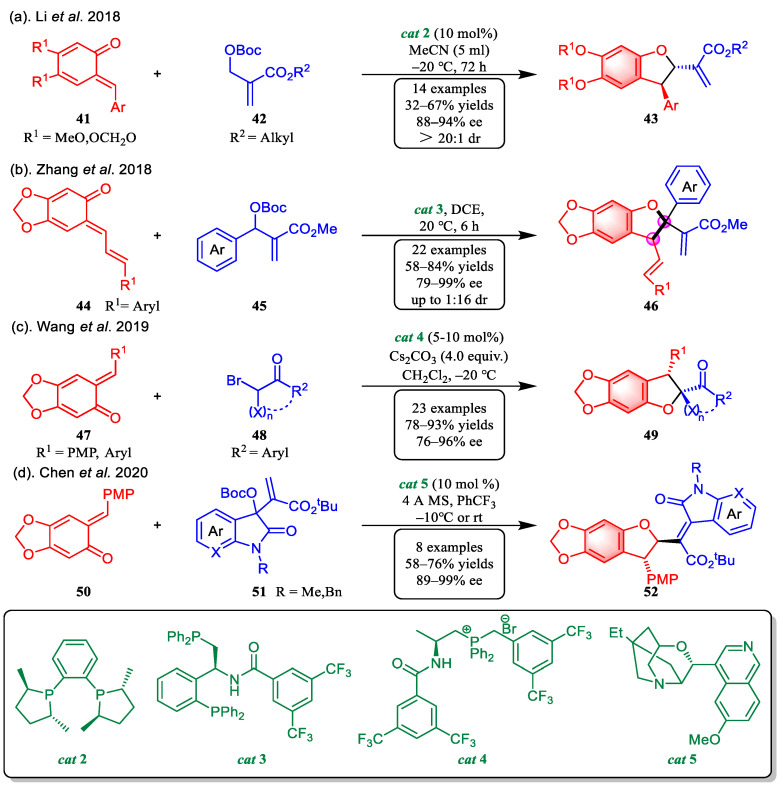
Asymmetric [4 + 1] cyclizations of *o*-QMs for the construction of chiral dihydrobenzofuran skeletons. (**a**) Li et al. 2018 [27]. (**b**) Zhang et al. 2018 [28]. (**c**) Wang et al. 2019 [29]. (**d**) Chen et al. 2020 [30].

Subsequent developments in 2018 further expanded asymmetric [4 + 1] cyclizations. Li et al. reported a chiral bisphosphine(**cat 2**)-catalyzed reaction of *o*-QMs **41** with MBH carbonates **42** to give dihydrobenzofurans **43** with excellent enantioselectivities (Scheme 14a) [27]. Electron-withdrawing groups gave lower yields than electron-donating ones. In situ generation of *o*-QMs failed. Compared with non-enantioselective methods, this approach achieved up to 94% ee, but substrate scope remains limited to MBH carbonates as C1 synthons.

Similarly, Zhang’s team developed a bifunctional chiral phosphine catalyst that enabled efficient cyclization under mild conditions with high stereocontrol. This strategy utilized an independently designed chiral bifunctional phosphine catalyst (**cat 3**) to achieve efficient cyclization of *o*-QMs **44** and MBH carbonates **45** at room temperature, successfully constructing a multi-substituted 2, 3-dihydrobenzofuran skeleton **46** (Scheme 14b) [28]. This reaction not only operates under mild conditions but also exhibits excellent yields and enantioselectivities, providing a precise and efficient approach for the synthesis of these important heterocyclic skeletons.

In 2019, Wang and co-workers introduced a bifunctional phosphonium salt (**cat 4**)-catalyzed asymmetric [4 + 1] cyclization of *o*-QMs **47** with α-bromoketones **48**, affording spiro-dihydrobenzofurans **49** in up to 93% yield, up to >20:1 dr, and 97% ee (Scheme 14c) [29]. The reaction proceeds in CH_2_Cl_2_ at −20 °C with Cs_2_CO_3_ as the base (4 equiv). Control experiments indicated that hydrogen-bonding interactions between the catalyst and substrates affect the stereoselectivity. Atom economy is moderate due to the stoichiometric base.

In 2020, Chen’s group demonstrated a **cat 5**-catalyzed regioselectivity-switchable strategy by tuning the steric effect of the ester group in MBH carbonates, enabling a switch from γ-[4 + 3] to α-[4 + 1] cyclization pathways (Scheme 14d) [30]. Using a chiral tertiary amine catalyst(**cat 5**), the reaction favors the [4 + 1] mode when MBH carbonates bear ethyl or benzyl esters, producing dihydrobenzofurans **52** in moderate yields (58–76%) with enantioselectivities up to 99% ee. This work illustrates how substrate fine-tuning can control reaction selectivity without additional catalyst modification.

**Scheme 15:**
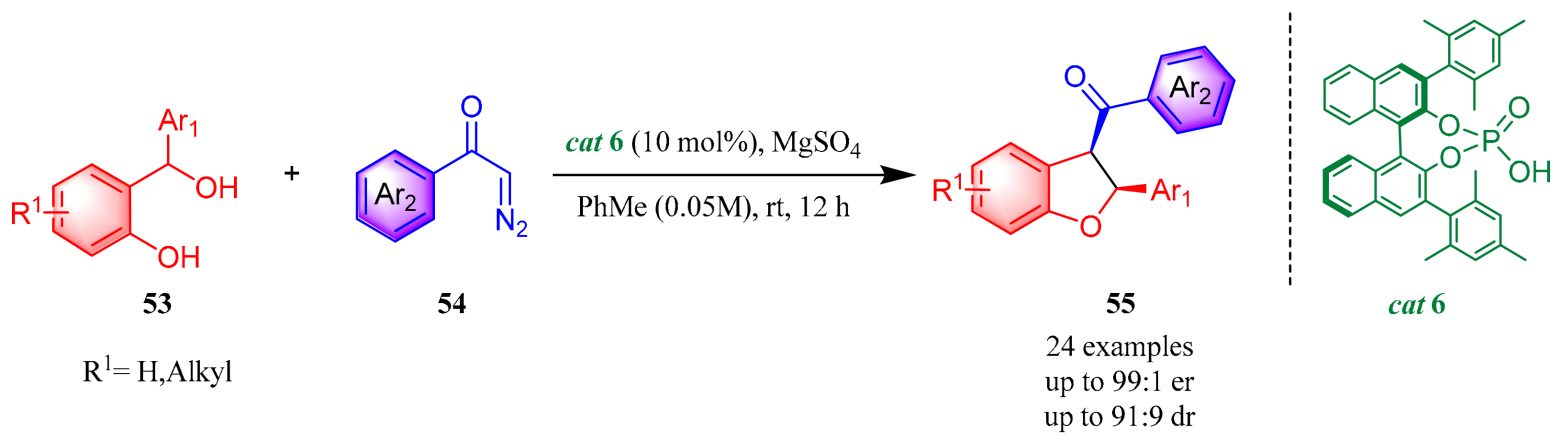
Phosphoric acid-catalyzed [4 + 1] cyclization of *o*-QMs with diazoketones.

In 2018, Schneider and co-workers developed a chiral Brønsted acid (**cat 6**)-catalyzed [4 + 1] cyclization of in situ-generated *o*-QMs **53** with α-diazoketones **54**, affording 2,3-dihydrobenzofurans **55** with an unusual 2,3-substitution pattern (aryl ketone at C3, PMP at C2) (Scheme 15) [31]. From the established typology, this transformation is a [4 + 1] cyclization. The reaction operates at room temperature, giving products in up to 91:9 dr and 99:1 er, and tolerates various substitution patterns. However, the substrate scope is limited to electron-rich β-methide substituents (e.g., PMP); alkyl or less electron-rich aryl groups give low or no yields. Atom economy is good as no stoichiometric additives are required beyond **cat 6** (10 mol%). Thus, from a cyclization perspective, this method provides a unique entry to cis-2,3-dihydrobenzofurans with inverted substitution, though the substrate scope restricts its broader applicability.

**Scheme 16:**
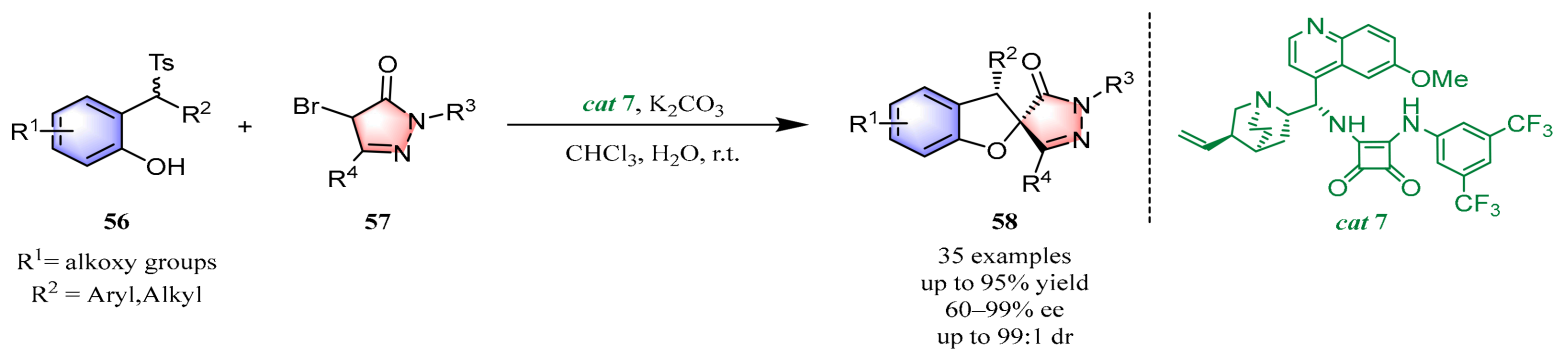
Amine-squaramide-catalyzed [4 + 1] cyclization of in situ-generated *o*-QMs with 4-halo pyrazolones.

In 2019, Xu and co-workers developed an asymmetric [4 + 1] cyclization of in situ-generated *o*-QMs with 4-halopyrazolones **57**, affording spiro-benzofuran pyrazolones **58** in a chloroform–water biphasic system using a chiral squaramide catalyst (**cat 7**) and K_2_CO_3_ as the base (Scheme 16) [32]. From the established typology, this transformation is a [4 + 1] cyclization proceeding via a conjugate addition/nucleophilic substitution sequence. The reaction tolerates a range of substituents on both coupling partners, delivering the products in 75–95% yield, with diastereoselectivities up to 99:1 dr and enantioselectivities up to 99% ee. A gram-scale experiment maintained the stereoselectivity. However, the method requires a biphasic solvent system and a stoichiometric base (K_2_CO_3_), which reduces operational simplicity. Atom economy is moderate due to the use of a stoichiometric base. Control experiments indicated that the choice of halogen (Br vs. Cl) influences the diastereoselectivity through different intramolecular substitution pathways, but detailed mechanistic studies were not provided. Thus, from a cyclization perspective, this protocol provides access to spiro-benzofuran pyrazolones with high stereocontrol, albeit with the requirement of a biphasic system and stoichiometric base.

**Scheme 17:**
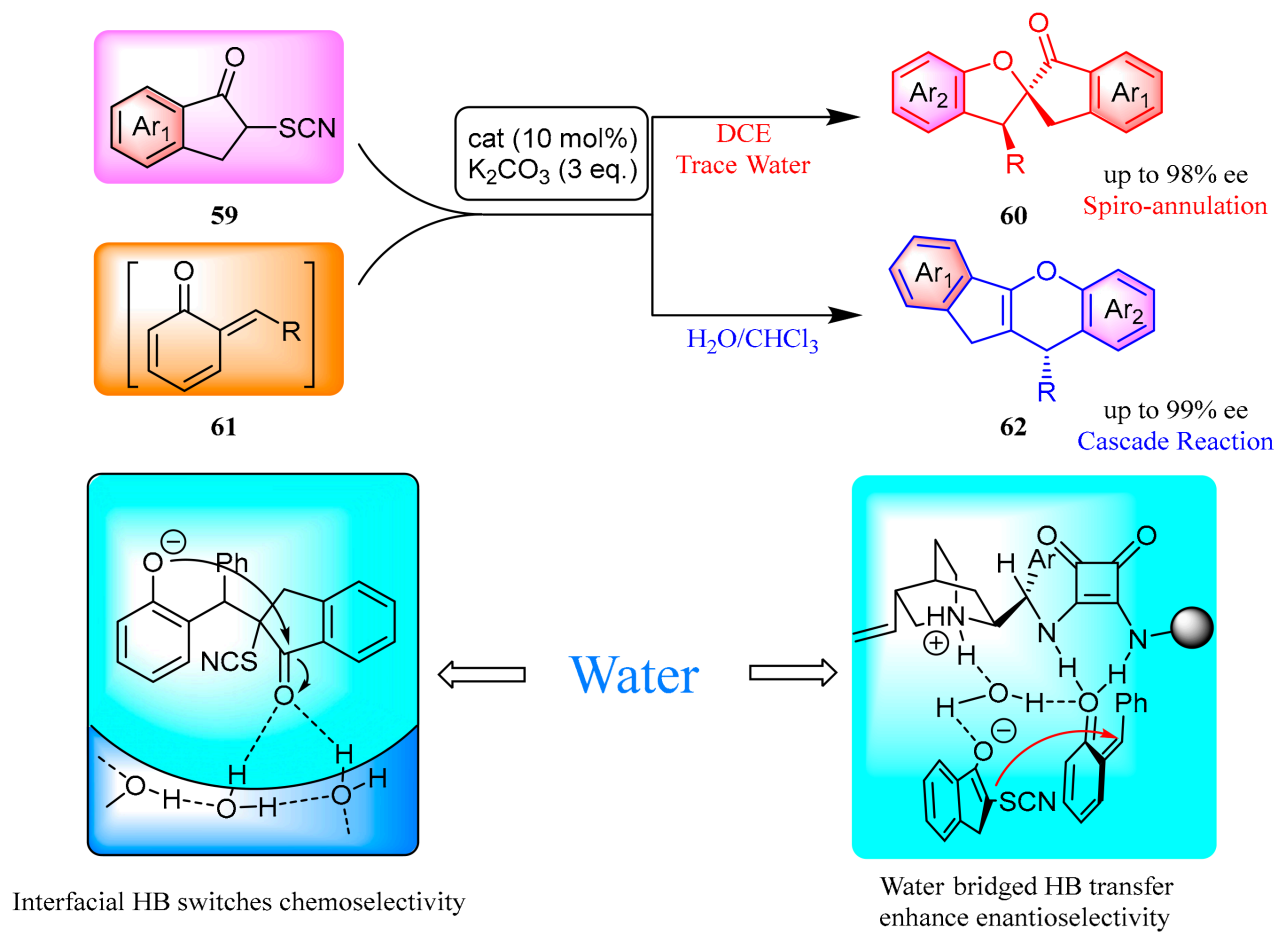
Asymmetric reactions of α-SCN-indanone with *o*-QMs.

In 2021, Li and co-workers developed a water-controlled chemoselective divergent synthesis of chiral indanone scaffolds using a single catalytic system (Scheme 17) [33]. From the established typology, this transformation involves a [4 + 1] cyclization pathway (spiro-annulation) and a cascade reaction (fused product), depending on the water content. The reaction proceeds via in situ-generated *o*-QMs **61** from 2-(tosylmethyl)phenol precursors, followed by asymmetric conjugate addition with α-thiocyanate indanones **59** catalyzed by a quinine-derived chiral squaramide. With trace water (5 equiv), the spirocyclic indanones **60** are obtained in up to 98% ee; with a water/chloroform (3:1) mixed solvent, the fused indanone derivatives **62** are formed in up to 99% ee. The method tolerates various substituents on both coupling partners, delivering the products in moderate to high yields (spiro products up to 90%, fused products up to 62% isolated yield). However, the cascade reaction gives only moderate yields, and both pathways require a stoichiometric base (K_2_CO_3_, 3 equiv) and long reaction times (48 h), which limit operational efficiency. Atom economy is moderate due to the use of a stoichiometric base. Thus, from a cyclization perspective, this work demonstrates a water-switchable divergent approach to two distinct indanone frameworks, albeit with the need for a stoichiometric base and relatively long reaction times.

## 3. [4 + 2]. Cyclization Reactions: The Dominant Paradigm

[4 + 2] cyclization represents the most extensively studied reaction mode of *ortho*-quinone methides (*o*-QMs), providing an efficient route to six-membered oxygen-containing heterocycles such as chromanes, dihydrocoumarins, and xanthenes. Owing to their inherent reactivity and well-matched electronic properties, *o*-QMs readily participate as 4π components in inverse electron-demand Diels–Alder-type processes. In recent years, significant progress has been achieved in the development of catalytic and asymmetric variants, enabling precise control over regio-, diastereo-, and enantioselectivity. These advances have established [4 + 2] cyclizations of *o*-QMs as a cornerstone strategy for the rapid construction of structurally complex and functionally diverse cyclic frameworks.

### 3.1. Transition-Metal-Free [4 + 2] Cyclization

**Scheme 18:**
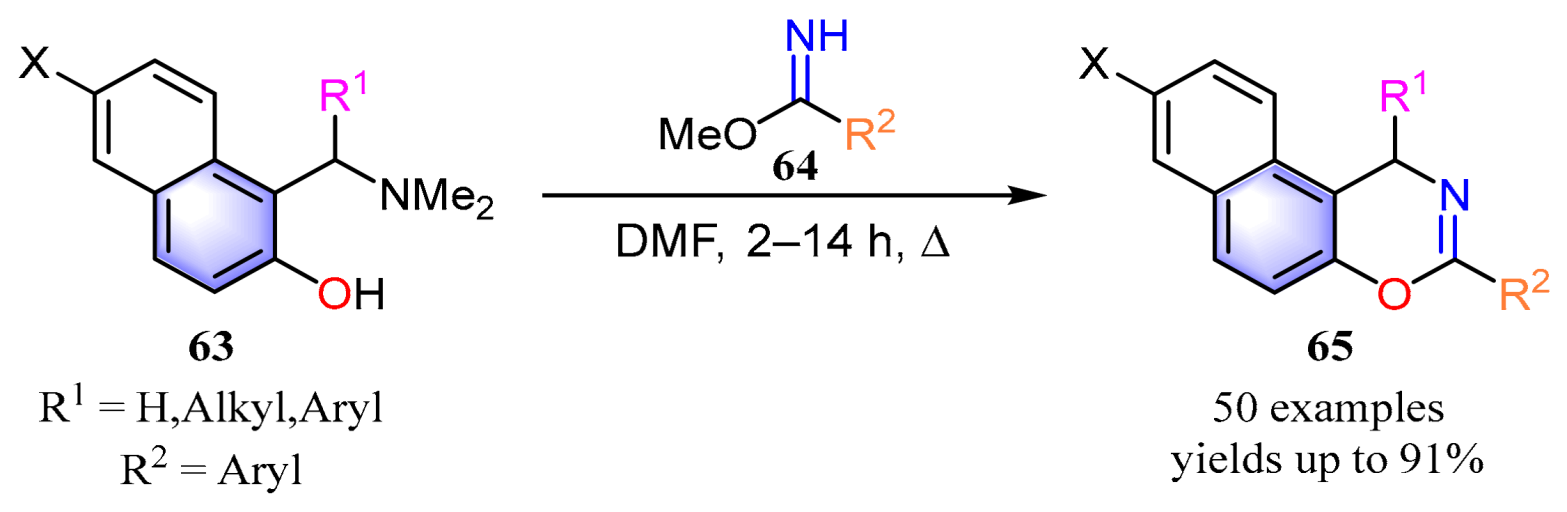
Catalyst-free inverse electron demands Azo-Diels-Alder reaction of *o*-QMs with imino ether.

In 2018, Osyanin et al. reported a catalyst-free formal [4 + 2] cycloaddition of *o*-QMs with imino ethers, which they described as an inverse electron-demand aza-Diels–Alder reaction (Scheme 18) [34]. From the established typology, this transformation provides a route to benzo-fused 1,3-oxazines. Mannich bases or 2-hydroxybenzyl alcohols **63** serve as *o*-QM precursors, reacting with imino ethers **64** followed by aromatization to afford products **65** in yields up to 91%. The method operates without any metal or organic catalyst, tolerates a range of substituents, and often avoids chromatographic purification. However, the reaction requires high temperature (DMF reflux, ca. 153 °C) and extended times (2–14 h). Some substrates give low yields, e.g., 4-chloro-substituted (22%) and 3-nitrophenyl derivative (24%). Atom economy is moderate due to the formation of dimethylamine and methanol as byproducts. Thus, from a cycloaddition perspective, this protocol offers a catalyst-free entry to benzoxazines, albeit with limited efficiency for certain substitution patterns.

**Scheme 19:**
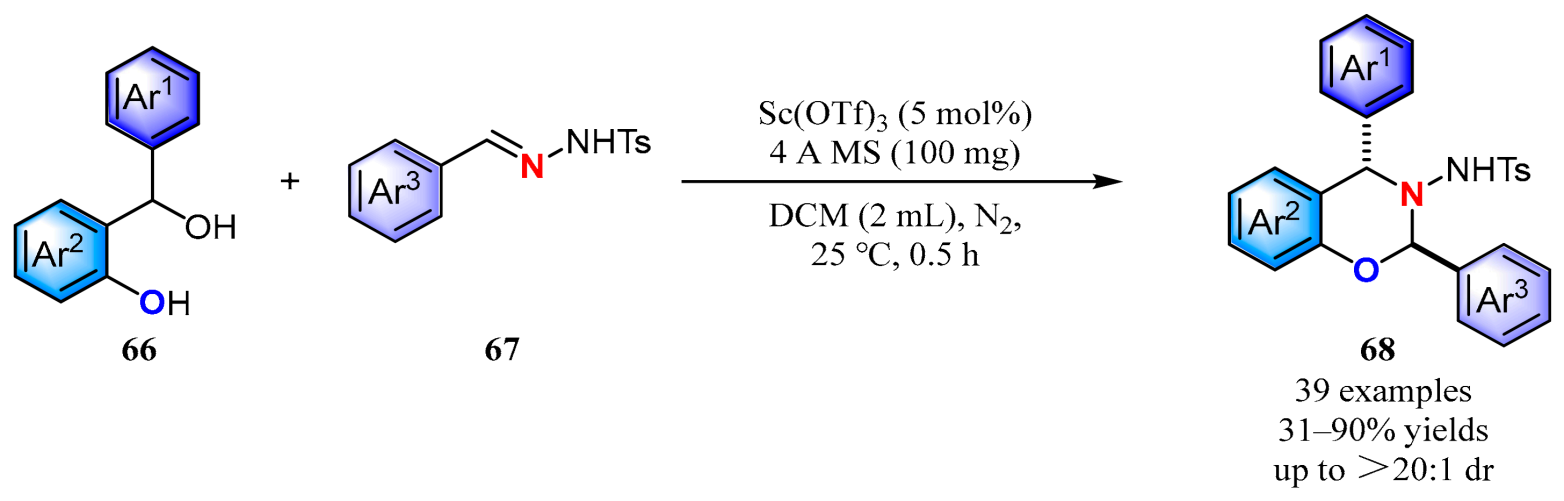
Lewis acid-catalyzed formal [4 + 2] cycloaddition of *N*-tosylhydrazones with *o*-QMs.

In 2019, Tang and co-workers developed a Lewis acid-catalyzed formal [4 + 2] cycloaddition of in situ-generated *o*-QMs with *N*-tosylhydrazones **67**, affording 3,4-dihydro-2H-benzo[e][1,3]oxazines **68** (Scheme 19) [35]. The reaction uses Sc(OTf)_3_ (5 mol%) at room temperature, giving products in 31–90% yield with up to >20:1 dr. The method tolerates various substituents, but yields are lower for ketone-derived hydrazones (34–79%) and a thienyl substrate (36%). Atom economy is moderate due to water as byproduct and excess hydrazone (1.3 equiv). Thus, from a cycloaddition perspective, this protocol offers a practical route to benzoxazines, albeit with limited efficiency for hindered substrates.

**Scheme 20:**
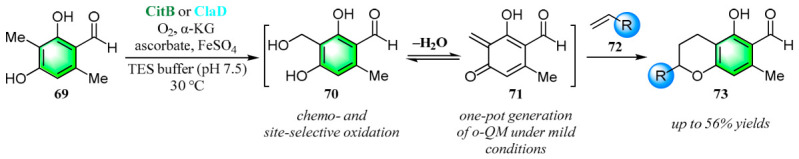
Chemoenzymatic generation of *o*-QMs for inverse electron-demand Diels–Alder formal [4 + 2] cycloadditions.

Also in 2019, Narayan and co-workers developed a chemoenzymatic cascade for the generation of *o*-QMs via non-heme iron enzyme-catalyzed benzylic C–H hydroxylation under mild, aqueous conditions (Scheme 20) [36]. The *o*-QMs bear an electron-withdrawing formyl group at C4 and undergo one-pot formal [4 + 2] cycloaddition with electron-rich dienophiles **72** (e.g., ethyl vinyl ether, styrenes) in an inverse electron-demand Diels–Alder fashion, affording chroman derivatives **73** in moderate to good yields (33–56%). However, the substrate scope is limited by the requirement for a hydrogen-bond acceptor at C4 of the phenol, and the biocatalyst preparation requires specialized expertise. Thus, from a cycloaddition perspective, the assignment of inverse electron demand is consistent with the electron-deficient *o*-QM (due to the formyl group) and the electron-rich dienophiles used.

**Scheme 21:**
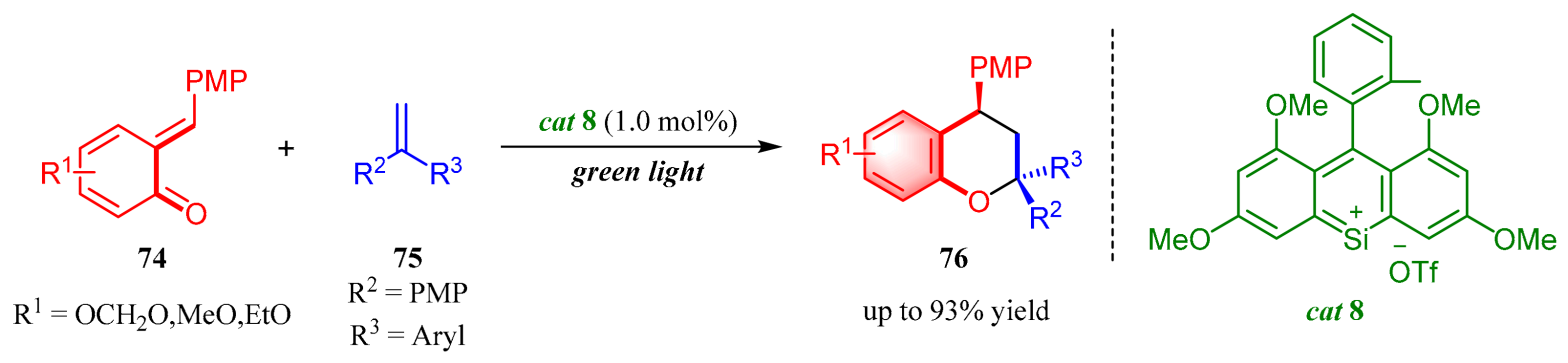
Research progress on photocatalytic *o*-QM formal oxa-[4 + 2] cycloaddition reactions.

In 2019, Honda, Hoshino, and co-workers reported a visible-light-driven intermolecular formal oxa-[4 + 2] cycloaddition between *o*-QMs **74** and alkenes **75** using a thioxanthylium photoredox catalyst (**cat 8**) under green light irradiation (Scheme 21) [37]. From the established typology, this transformation represents a formal [4 + 2] cycloaddition proceeding via radical cation intermediates. The reaction affords dihydrochromene derivatives 76 in moderate to good yields (up to 93%). The method operates at room temperature with a catalytic amount of photocatalyst (1–5 mol%) and uses visible light as the energy source, avoiding thermal activation. However, the substrate scope is largely limited to electron-rich styrenes (e.g., those bearing methoxy groups); styrene itself and electron-deficient alkenes give low or no yields. The products are obtained as racemic mixtures, and the reaction requires an excess of *o*-QM (3 equiv relative to alkene), which reduces atom economy. Thus, from a cycloaddition perspective, this work provides the first example of a visible-light-induced intermolecular oxa-[4 + 2] cycloaddition involving *o*-QMs, but the narrow substrate scope and stoichiometric excess of one component limit its synthetic utility.

**Scheme 22:**
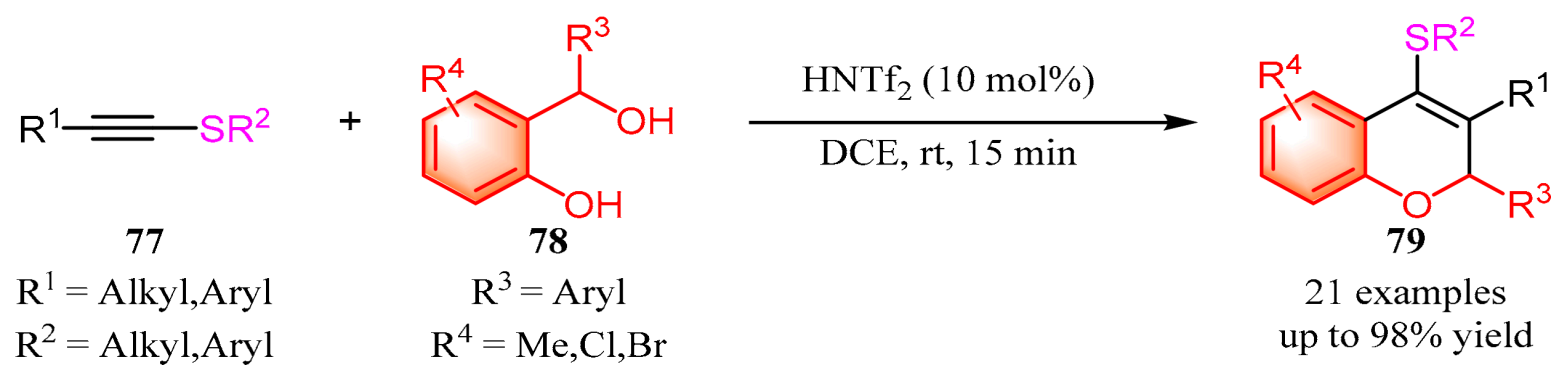
Brønsted acid-catalyzed [4 + 2] cyclization of alkynyl thioethers with *o*-hydroxybenzyl alcohols.

In 2020, Ye and co-workers reported a Brønsted acid-catalyzed [4 + 2] cyclization of alkynyl thioethers **77** with *o*-hydroxybenzyl alcohols **78**, affording polysubstituted 2*H*-chromenes **79** in good to excellent yields (60–98%) under mild conditions (Scheme 22) [38]. From the established typology, this transformation represents a [4 + 2] cyclization. The reaction features a broad substrate scope, good functional group tolerance, and can be performed on a gram scale (75% yield at 5 mol% catalyst loading). Accordingly, from a cyclization perspective, this protocol provides a metal-free and practical route to valuable 2H-chromenes with high efficiency.

**Scheme 23:**
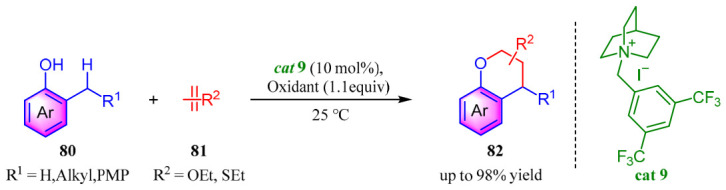
Hypoiodite-catalyzed chemoselective oxidative generation of *o*-QMs for formal [4 + 2].

Also in 2020, Uyanik, Ishihara and co-workers developed a **cat 9**-catalyzed chemoselective oxidation of *o*-alkylphenols **80** to generate *o*-QMs under mild, transition-metal-free conditions (Scheme 23) [39]. From the established typology, the in situ-generated *o*-QMs can undergo various tandem transformations, including formal [4 + 2] cycloaddition with electron-rich dienophiles **81** (e.g., ethyl vinyl ether) to afford chroman derivatives **82** in high yields (up to 99%). The method uses inexpensive hydrogen peroxide (30% aq.) or cumene hydroperoxide as the terminal oxidant, features a broad substrate scope and good functional group tolerance, and can be performed on a gram scale. It was successfully applied to the synthesis of the natural product (±)-schefflone (72% yield). As summarized in Figure 6a of the original report [39], mechanistic studies—including kinetic isotope effects (KIE = 1.8) and radical-trapping experiments—supported a hypoiodite-mediated oxidative pathway involving β-elimination to generate the *o*-QMs. The catalytic system requires an iodide salt (10 mol%) and an ammonium or sodium counterion, and is limited to electron-rich phenols or naphthols; less electron-rich substrates gave complex mixtures. Atom economy is moderate due to the use of a stoichiometric oxidant (H_2_O_2_ or CHP, 1.1 equiv). Thus, from a cycloaddition perspective, this protocol provides a sustainable, metal-free entry to oxygen heterocycles via oxidative *o*-QM generation, with a well-supported mechanistic rationale.

**Scheme 24:**
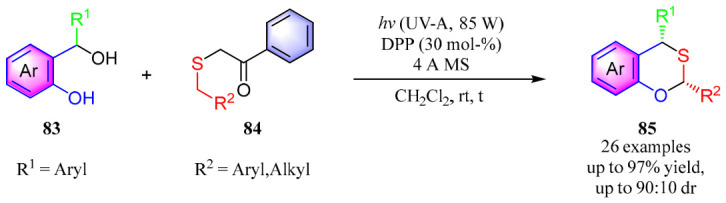
Cooperative photochemical/Bronsted acid-catalyzed formal [4 + 2] cycloaddition of thioaldehydes with *o*-QMs.

In 2021, Schneider and co-workers reported a cooperative photochemical/Brønsted acid-catalyzed formal [4 + 2] cycloaddition between thioaldehydes and *o*-QMs (Scheme 24) [40]. From the established typology, the thioaldehydes are generated in situ from phenacyl sulfides **84** under UV-A light (365 nm), while the *o*-QMs are produced from benzhydryl alcohols **83** via Brønsted acid (diphenyl phosphate, 10 mol%)-catalyzed dehydration. The two transient intermediates undergo an inverse electron-demand thia-Diels–Alder cycloaddition to afford benzo[e][1,3]oxathine S,O-heterocycles **85** in good to excellent yields (35–97%) with moderate to good diastereoselectivity (up to 90:10 dr). The reaction proceeds at room temperature in short reaction times (6 h) under dilute conditions (0.01 M). However, the substrate scope is limited: electron-withdrawing substituents on either the thioaldehyde or the *o*-QM significantly reduce yields (e.g., a chloro-substituted benzoxathine in 35% yield and an ester-substituted analogue not formed), and alkyl thioaldehydes also give lower yields (e.g., 35%). The method requires UV irradiation and a Brønsted acid, which may limit practicality for large-scale applications. Atom economy is moderate due to the use of a stoichiometric phenacyl sulfide precursor and the generation of acetophenone as a byproduct. Thus, from a cycloaddition perspective, this work represents the first example of a thia-Diels–Alder reaction involving *o*-QMs, providing a metal-free route to S,O-heterocycles, albeit with substrate constraints.

**Scheme 25:**
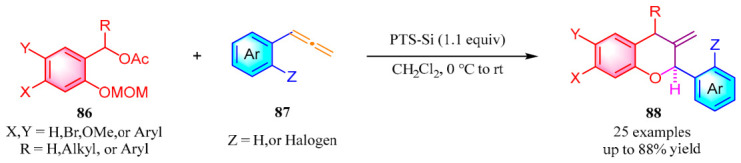
PTS-Si-mediated formal [4 + 2] cycloaddition of *o*-QMs with arylallenes for the synthesis of functionalized 3-methylenechromans.

In 2022, Ploypradith and co-workers reported a PTS-Si-mediated formal [4 + 2] cycloaddition of *o*-QM precursors **86** with arylallenes **87**, affording 3-methylene-2-arylchromans **88** in moderate to good yields (30–88%) (Scheme 25) [41]. From the established typology, this transformation represents a formal [4 + 2] cycloaddition. The stereochemistry (cis or trans) of the C2–C4 bond is governed by the benzylic substituent on the *o*-QM precursor: a methyl group favors the cis isomer, while an aryl group favors the trans isomer. The method tolerates a range of substituents on both coupling partners and can be performed on a gram scale. The products serve as versatile intermediates for further functionalizations, including epoxidation, Riley oxidation, acid-catalyzed rearrangement, and Pd-catalyzed cross-coupling. However, the substrate scope is limited: *o*-QM precursors lacking an electron-donating group on the phenol ring give no product, and some arylallenes afford low yields (e.g., a bromo-substituted allene gave 30% yield). The reaction requires an excess of arylallene (2 equiv) and a stoichiometric amount of PTS-Si (1.1 equiv), which reduces atom economy. Thus, from a cycloaddition perspective, this protocol provides a stereodivergent entry to functionalized chromans with useful synthetic handles, albeit with certain substrate restrictions and moderate atom economy.

**Scheme 26:**
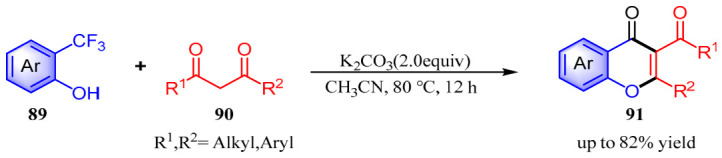
Defluorinative [4 + 2] cyclization of difluor *o*-QMs with 1,3-Ddicarbonyls for chromone synthesis and plausible mechanism.

More recently, in 2025, Dhiman and co-workers developed a transition-metal-free defluorinative [4 + 2] cyclization of 2-trifluoromethylphenol **89** with 1,3-dicarbonyl compounds **90**, affording 3-acyl chromones **91** in moderate to good yields (up to 82% yield) (Scheme 26) [42]. From the established typology, this transformation represents a [4 + 2] cyclization. The reaction proceeds under mild conditions using K_2_CO_3_ as the base, and can be performed on a gram scale (77% yield). The substrate scope includes a range of 1,3-diketones, β-ketoesters, and substituted 2-trifluoromethylphenols, tolerating halogen, aromatic, heteroaromatic, and vinyl substituents. However, β-ketoamides gave no product, and a strongly electron-withdrawing group on the phenol ring prevented the reaction. The use of a stoichiometric base (2 equiv K_2_CO_3_) and an excess of the dicarbonyl compound (2 equiv) reduces atom economy. Thus, from a cyclization perspective, this protocol offers a metal-free route to functionalized chromones, albeit with moderate atom economy and some substrate limitations.

**Scheme 27:**
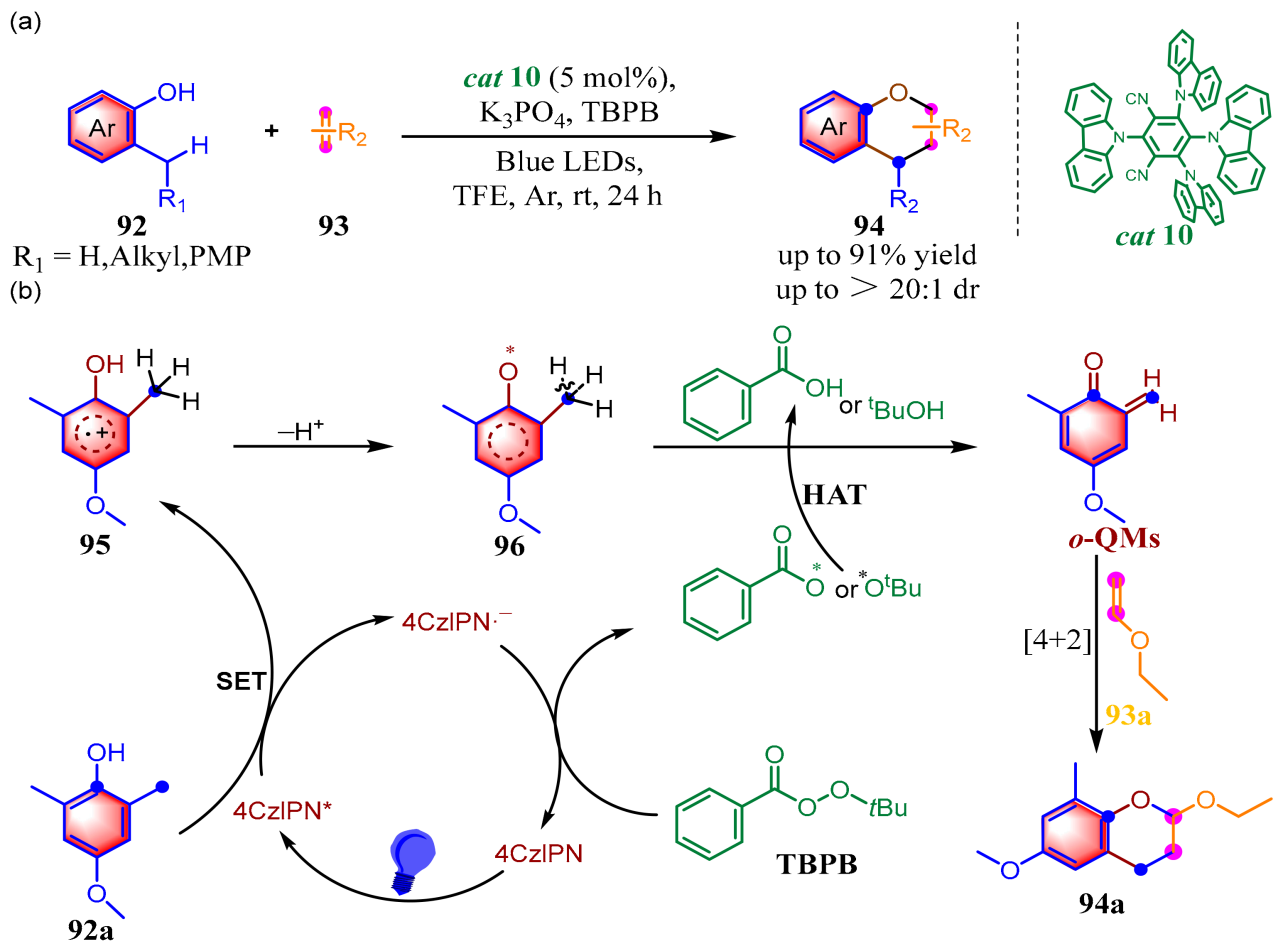
(**a**) Visible-light-induced generation of *o*-QMs from *ortho*-alkylarenols for tandem formal [4 + 2] cycloaddition and (**b**) the possible mechanism.

In the same year, Wu and co-workers developed a visible-light-induced, metal-free oxidative generation of *o*-QMs from *ortho*-alkylarenols **92**, followed by a formal [4 + 2] cycloaddition with alkenes **93** to afford chromans **94** in moderate to good yields (45–91%) (Scheme 27a) [43]. From the established typology, this transformation represents a formal [4 + 2] cycloaddition. The reaction proceeds under mild conditions (room temperature, blue LED) using **cat 10** (4CzIPN) as the photocatalyst, K_3_PO_4_ as the base, and tert-butyl peroxybenzoate (TBPB) as the oxidant. The substrate scope includes a range of *ortho*-alkylarenols and various alkenes (monosubstituted, 1,1-disubstituted, and 1,2-disubstituted); a gram-scale reaction gave 72% yield. However, the reaction requires a large excess of alkene (15 equiv) and a stoichiometric oxidant (1.4 equiv TBPB), which reduces atom economy. Electron-deficient arenes gave only trace products. Mechanistic studies, including radical trapping experiments (TEMPO suppressed the reaction), kinetic isotope effect (kH/kD = 3.8), and Stern-Volmer quenching, supported the proposed pathway outlined in Scheme 27b: single-electron transfer (SET) from the substrate to the excited photocatalyst generates a radical cation, which undergoes deprotonation and hydrogen atom transfer (HAT) to form the *o*-QM. The *o*-QM then participates in a formal inverse electron-demand [4 + 2] cycloaddition with electron-rich alkenes to give the chroman products with good diastereoselectivity (up to >20:1 dr). Thus, from a cycloaddition perspective, this protocol offers a metal-free, visible-light-driven route to chromans, albeit with moderate atom economy and a requirement for excess alkene.

**Scheme 28:**
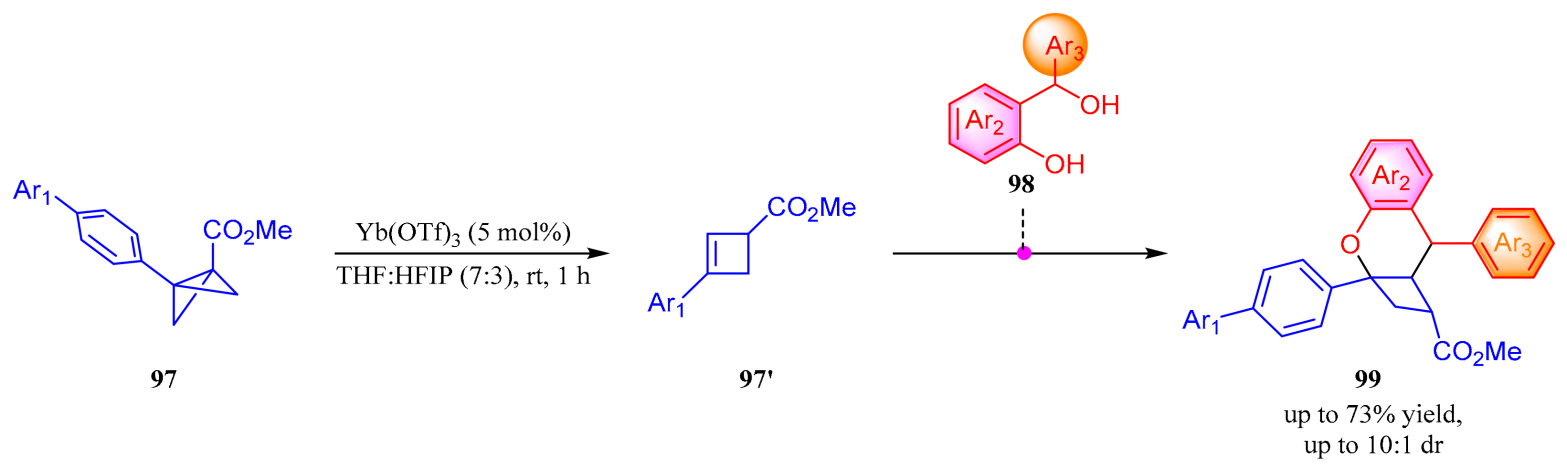
The divergent formal [4 + 2] cycloaddition of *o*-QMs enables the construction of oxygen-bridged bicyclic skeletons.

In 2026, Saha and co-workers reported a Lewis acid-catalyzed divergent annulation of *o*-QMs (generated in situ from 2-hydroxybenzyl alcohols **98**) with bicyclo[1.1.0]butanes **97** (Scheme 28) [44]. From the established typology, the reaction pathway can be switched between formal [4 + 3] and formal [4 + 2] cycloadditions by varying the reaction conditions. Under Yb(OTf)_3_ catalysis in a THF/HFIP (7:3) solvent mixture, the BCB undergoes direct formal [4 + 3] cycloaddition with the *o*-QM to give oxabicyclo[4.1.1]octanes. Alternatively, under similar conditions but with a different solvent composition, the BCB first isomerizes to a cyclobutene, which then engages in a formal [4 + 2] cycloaddition with the *o*-QM to afford 2-oxabicyclo[4.2.0]octanes **99** in moderate to good yields (56–73%) with high regioselectivity. However, the reaction requires a Lewis acid catalyst (5 mol% Yb(OTf)_3_) and a mixed solvent system, and the yields for the [4 + 2] pathway are modest. Atom economy is moderate due to the use of a stoichiometric excess of the *o*-QM precursor (1.5 equiv) and the catalyst. Thus, from a cycloaddition perspective, this protocol provides a switchable entry to sp^3^-rich oxabicyclic frameworks, albeit with moderate yields.

### 3.2. Transition-Metal-Catalyzed [4 + 2] Cyclization

**Scheme 29:**
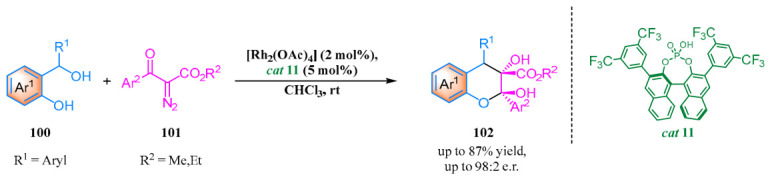
Synergistic Rhodium/Brønsted acid-catalyzed enantioselective [4 + 2] cyclization of oxonium ylides with *o*-QMs.

Transition-metal catalysis has expanded the scope of [4 + 2] cyclizations involving *o*-QMs. In 2016, Schneider and co-workers reported a synergistic rhodium/phosphoric acid catalytic strategy for an enantioselective [4 + 2] cyclization of in situ-generated oxonium ylides with *o*-QMs (Scheme 29) [45]. From the established typology, this transformation proceeds via a tandem conjugate addition–hemiacetalization sequence rather than a concerted cycloaddition. The reaction furnishes chromans **102** bearing three contiguous stereocenters in moderate to good yields (55–87%) with excellent enantioselectivities (up to 98:2 er). Mechanistically, phosphoric acid generates the *o*-QM from precursor **100**, while the water byproduct reacts with a rhodium carbene to form the oxonium ylide, which then undergoes conjugate addition and hemiacetalization. However, the dual catalyst system (5 mol% **cat 11**, 2 mol% Rh_2_(OAc)_4_) and slow diazoester addition limit operational simplicity. Atom economy is moderate due to stoichiometric excess of diazoester (1.2 equiv) and water byproduct. Thus, from a cyclization perspective, this synergistic approach provides a powerful entry to densely functionalized chromans, albeit with some complexity.

**Scheme 30:**
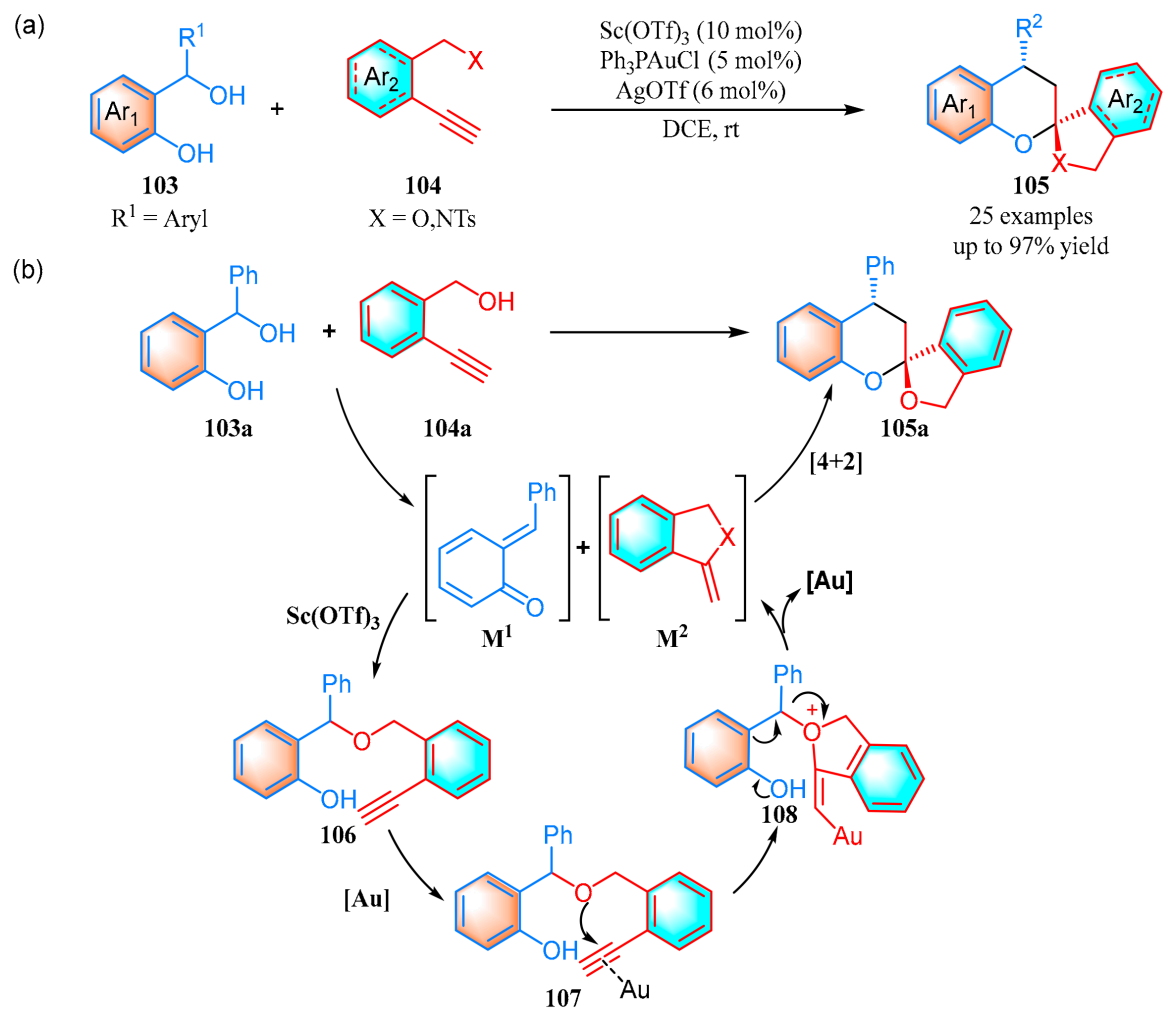
(**a**) Synergistic gold/scandium-satalyzed formal [4 + 2] cycloaddition for spiroketal synthesis and (**b**) plausible mechanism.

In 2017, Xu and co-workers developed a synergistic Au(I)/Sc(III) catalytic system for a formal [4 + 2] cycloaddition of o-hydroxybenzyl alcohols **103** with alkynyl alcohols **104**, enabling the rapid construction of 5,6-benzo-fused spiroketals **105** in high yields (up to 97%) (Scheme 30a) [46]. From the established typology, this transformation represents a formal [4 + 2] cycloaddition. The reaction proceeds at room temperature within 5 min, features broad substrate scope, and is scalable (0.5 mol% Au catalyst gave 65% yield on a 4 mmol scale). Mechanistically, Sc(OTf)_3_ promotes dehydration of 103a to generate an electrophilic *o*-QM (M^1^), while the gold catalyst activates 104a to undergo 5-exo-dig cyclization, forming a nucleophilic enol ether (M^2^). These intermediates then undergo a direct [4 + 2] cycloaddition to give the spiroketal. Control experiments support an alternative pathway as well: initial Sc-catalyzed conjugate addition produces an acyclic intermediate, which is converted to the same product under gold catalysis. However, the reaction requires two different metal catalysts (5 mol% Au and 10 mol% Sc) and an excess of the alkynyl alcohol (1.2 equiv), which reduces atom economy. Thus, from a cycloaddition perspective, this synergistic approach provides an ultrafast, practical route to complex spiroketals, albeit with moderate atom economy and dual-metal catalysis.

**Scheme 31:**
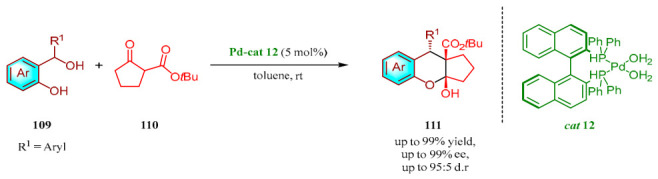
Cooperative palladium-catalyzed enantioselective [4 + 2] cyclization of cyclic β-keto esters with *o*-QMs.

In 2018, Schneider and co-workers developed a synergistic chiral palladium-catalyzed enantioselective [4 + 2] cyclization of in situ-generated *o*-QMs with cyclic β-keto esters **110** (Scheme 31) [47]. From the established typology, this transformation represents a [4 + 2] cyclization proceeding via a conjugate addition/hemiacetalization cascade. The method efficiently constructs highly functionalized chroman scaffolds **111** bearing two adjacent quaternary stereocenters and one tertiary stereocenter, achieving excellent yields (up to 99%), high diastereoselectivity (up to 95:5 d.r.), and outstanding enantioselectivity (up to 99% ee). Mechanistically, the chiral Pd–aqua complex (e.g., **cat 12**) acts as a bifunctional Brønsted acid/base catalyst. The acidic component promotes dehydration of benzhydryl alcohol **109** to generate the *o*-QM, while the conjugate base deprotonates the β-keto ester **110** to form a nucleophilic palladium enolate. These intermediates undergo a highly stereoselective conjugate addition via an open transition state, followed by hemiacetalization and cyclization. The reaction requires a palladium catalyst (5 mol%) and proceeds at room temperature without additional base, which is atom economical. However, the use of a precious metal and a chiral bisphosphine ligand may limit large-scale applications. Nevertheless, from a cyclization perspective, this cooperative Pd/Brønsted acid catalysis provides a powerful and stereodivergent route to densely functionalized chromans, complementing previously reported phosphoric acid-based strategies.

**Scheme 32:**
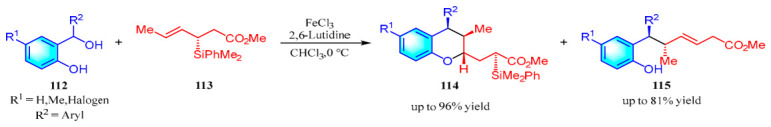
FeCl_3_-mediated enantioselective formal [4 + 2] cycloaddition and 1,4-addition of *o*-QMs with chiral crotyl silane.

In 2019, Panek and co-workers reported an FeCl_3_-promoted enantioselective strategy enabling both formal [4 + 2] cycloaddition and 1,4-addition reactions between diols **112** and enantiomerically enriched (*S,E*)-crotyl silane **113** (Scheme 32) [48]. From the established typology, this transformation proceeds through in situ generation of *o*-QMs, which can undergo either formal inverse electron-demand [4 + 2] cycloaddition to give chiral chromans **114** or 1,4-conjugate addition to afford crotylated products **115**. The product ratio is governed by the electronic nature of the *o*-QM: electron-rich systems favor cycloaddition, while electron-deficient substrates favor 1,4-addition. The method delivers mixtures of both products in high combined yields (up to 96%) and with good diastereoselectivity (up to 22:1 dr). The reaction uses inexpensive FeCl_3_ (0.8 equiv) and 2,6-lutidine (0.4 equiv) as additives, operates at room temperature, and tolerates a range of substituents. However, the need for a stoichiometric amount of FeCl_3_ and the formation of product mixtures reduce atom economy and require additional purification. Nevertheless, from a cycloaddition perspective, this work highlights the dual reactivity of allyl silanes and significantly expands their utility in asymmetric synthesis, offering a complementary approach to both chroman and crotylated frameworks.

**Scheme 33:**
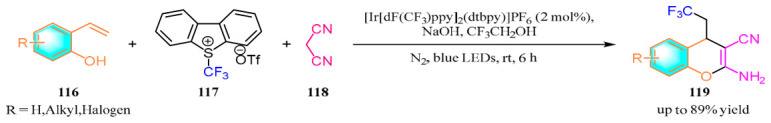
Visible-light photocatalytic [4 + 2] cyclization cascade for 4*H*-chromene synthesis.

In 2021, Kim and co-workers developed a visible-light-driven three-component cascade reaction of 2-vinyl phenols **116**, Umemoto’s reagent **117**, and malononitrile **118** for the efficient synthesis of trifluoromethylated 4H-chromenes **119** (Scheme 33) [49]. From the established typology, this transformation represents a [4 + 2] cyclization proceeding via in situ generation of an *o*-QM intermediate. The method affords the desired products in moderate to good yields (up to 89% yield) under mild, visible-light conditions, and tolerates a range of substituents on the vinyl phenol. However, the reaction requires a stoichiometric amount of Umemoto’s reagent (1.2 equiv) and a base (NaOH, 2 equiv), which reduces atom economy. The use of an expensive iridium photocatalyst (2 mol%) may also limit scalability. Thus, from a cyclization perspective, this protocol provides a concise route to trifluoromethylated chromenes, albeit with moderate atom economy and reliance on a precious metal photocatalyst.

### 3.3. Asymmetric Catalysis in [4 + 2] Cyclization

**Scheme 34:**
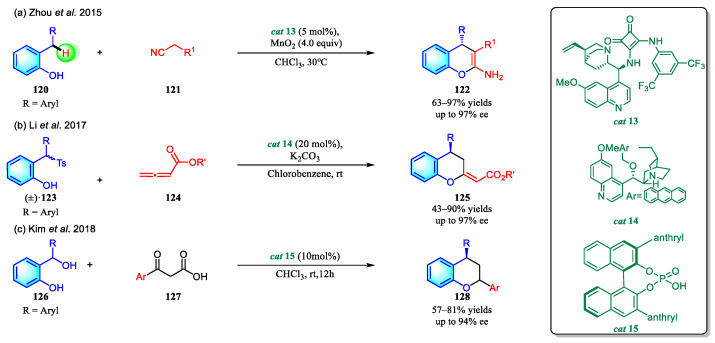
Chiral catalysis controls active intermediates: three strategic evolutions of *o*-QM asymmetric transformation. (**a**) Zhou et al. 2015 [50]. (**b**) Li et al. 2017 [51]. (**c**) Kim et al. 2018 [52].

In 2015, Zhou et al. reported a tandem C-H oxidation/Michael addition/cyclization for enantioselective synthesis of 2-amino-4*H*-chromenes **122** from 2-alkylphenols **120** and malononitrile **121** (Scheme 34a) [50]. From the established typology, this [4 + 2] cyclization proceeds via in situ generation of an *o*-QM intermediate using MnO_2_ as the oxidant and a chiral squaramide **cat 13** as the catalyst. The method gives good yields (up to 97%) and high enantioselectivities (up to 97% ee) with broad substrate scope. However, the use of stoichiometric MnO_2_ (4 equiv) reduces atom economy. Thus, from a cyclization perspective, this cascade provides an efficient route to chiral chromenes, albeit with moderate atom economy.

In 2017, Li et al. developed enantioselective transformations of 2-sulfonylalkyl phenols **123** based on reversible in situ generation of *o*-QMs (Scheme 34b) [51]. From the established typology, a formal [4 + 2] cycloaddition pathway operates when using a chiral cinchona-derived catalyst **cat 14** and K_2_CO_3_ as the base, affording 4-substituted chromans **125** in excellent enantioselectivities (86–97% ee). The reaction tolerates various substituents and proceeds under mild conditions. However, the cycloaddition requires a stoichiometric base (2 equiv K_2_CO_3_) and a relatively high catalyst loading (20 mol%), which reduces atom economy. Thus, from a cycloaddition perspective, this work provides a catalyst-switchable entry to enantiomerically enriched chromans, albeit with moderate atom economy.

In 2018, Kim’s group developed a one-pot, two-step enantioselective decarboxylative alkylation/cyclization/dehydration sequence of β-keto acids **127** with in situ-generated *o*-QMs (from **126**) for the synthesis of chiral 2,4-diaryl-1-benzopyrans **128** (Scheme 34c) [52]. From the established typology, this transformation represents a [4 + 2] cyclization. The reaction uses **cat 15** (10 mol%) and Sc(OTf)_3_ (50 mol%) as additives, affording products in moderate to good yields (57–81%) and high enantioselectivities (76–94% ee). The method tolerates a range of substituents and is scalable (gram-scale, 78% yield, 91% ee). However, the need for a stoichiometric amount of Sc(OTf)_3_ (0.5 equiv) and a relatively high catalyst loading reduces atom economy. Thus, from a cyclization perspective, this protocol provides an efficient route to chiral benzopyrans, albeit with moderate atom economy.

From the established typology, the following formal [4 + 2] cycloadditions of *o*-QMs with azlactones represent formal cycloadditions leading to chiral dihydrocoumarins.

**Scheme 35:**
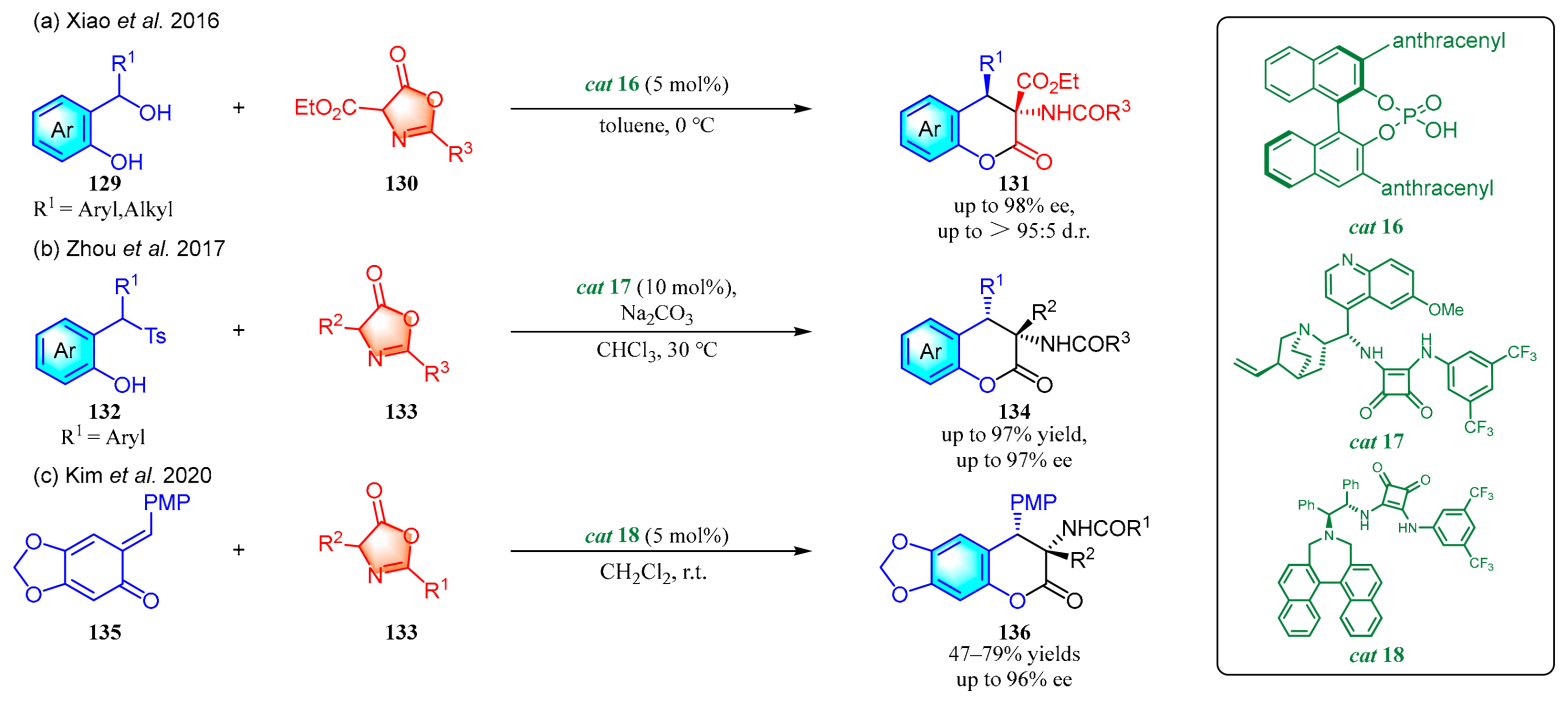
From triple activation to hydrogen-bonding catalysis: methodological evolution of the cycloaddition reaction between *o*-QMs and azlactone. (**a**) Xiao et al. 2016 [53]. (**b**) Zhou et al. 2017 [54]. (**c**) Kim et al. 2020 [55].

In 2016, Xiao reported a chiral phosphoric acid-catalyzed formal [4 + 2] cycloaddition between *o*-QM precursor **129** and azlactone **130** via a triple Brønsted acid activation mode (**cat 16**), affording products **131** in good yields (up to 94%) and high enantioselectivities (up to 98% ee) (Scheme 35a) [53]. However, the reaction required a stoichiometric amount of 4 Å molecular sieves and a relatively high catalyst loading (5 mol%).

In 2017, Zhou employed a bifunctional squaramide catalyst (***cat 17***) to promote a formal [4 + 2] cycloaddition of *o*-QM intermediates (from **132**) with azlactones **133** under mild conditions (30 °C) in a water–oil biphasic medium (Scheme 35b) [54]. This system expanded the substrate scope to include alkyl-substituted azlactones and gave products **134** in moderate to good yields (up to 97% yield) with excellent enantioselectivities (up to 97% ee). The biphasic system eliminated the need for molecular sieves, albeit with a higher catalyst loading.

In 2020, Kim developed a binaphthyl-modified squaramide catalyst (**cat 18**) for the reaction of stable *o*-QMs **135** with azlactones, affording products 136 at room temperature with 5 mol% catalyst loading and excellent enantioselectivities (up to 96% ee) (Scheme 35c) [55]. This protocol further simplified reaction conditions by using stable *o*-QMs but still required a stoichiometric base (K_2_CO_3_, 2 equiv), limiting atom economy.

Thus, from a cycloaddition perspective, these studies demonstrate progressive improvements, from the establishment of a triple-activation mode (Xiao) to expansion of substrate scope under mild biphasic conditions (Zhou), and finally to the use of stable *o*-QMs at room temperature with low catalyst loading (Kim). However, all methods rely on stoichiometric additives (molecular sieves or base), resulting in moderate atom economy.

**Scheme 36:**
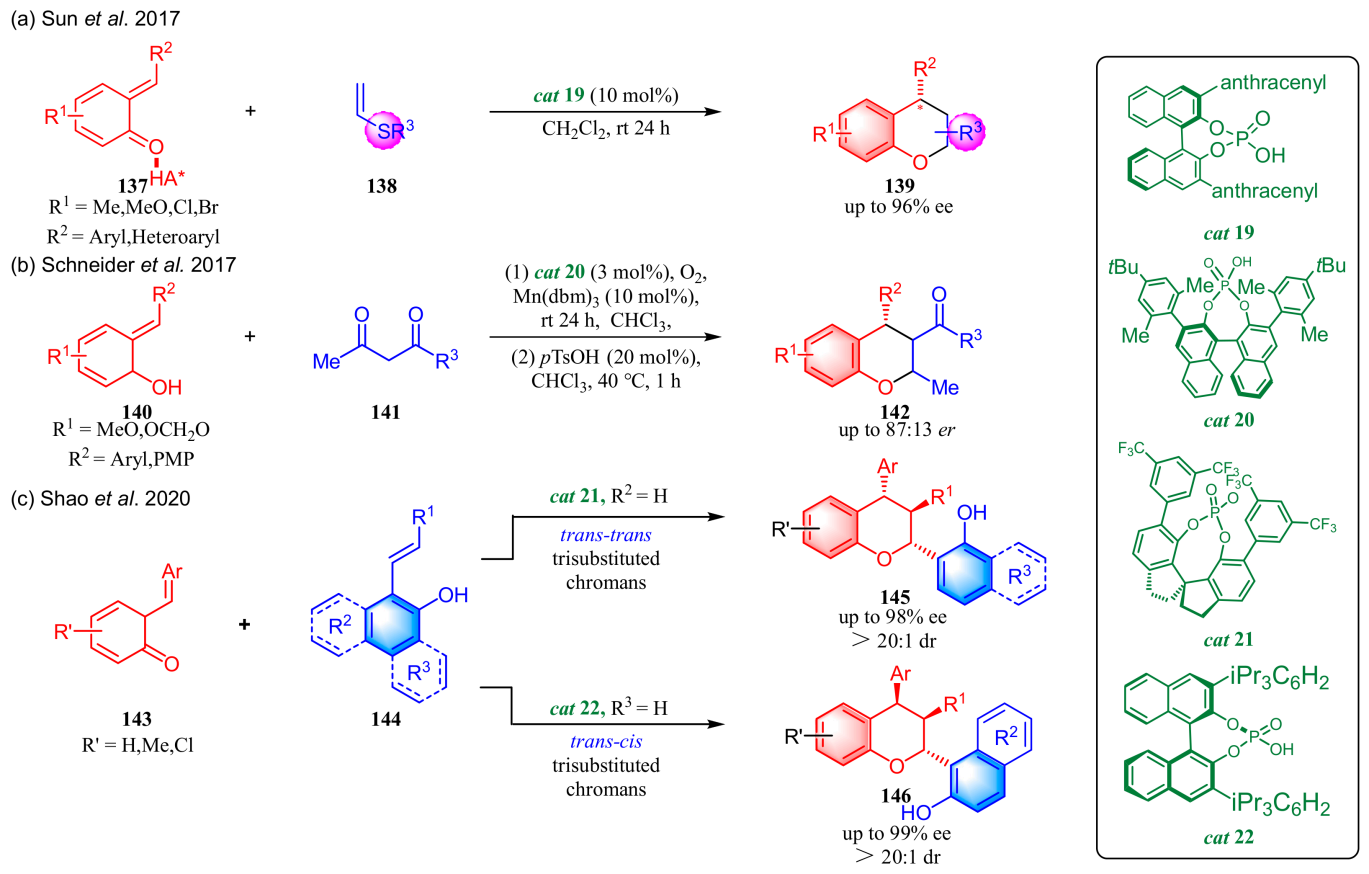
From single activation to relay catalysis: methodological evolution of asymmetric formal [4 + 2] cycloaddition reactions involving *o*-QMs. (**a**) Sun et al. 2017 [56]. (**b**) Schneider et al. 2017 [57]. (**c**) Shao et al. 2020 [58].

In the field of asymmetric cycloaddition reactions of *o*-quinone methides (*o*-QMs), overcoming substrate limitations and developing innovative catalytic modes have remained central research challenges. In 2017, Sun et al. reported a chiral phosphoric acid (**cat 19**)-catalyzed formal [4 + 2] cycloaddition of in situ-generated *o*-QMs **137** with vinyl sulfides **138**, affording chiral chromans **139** with excellent diastereoselectivity (>30:1 dr) and good enantioselectivity (up to 96% ee) (Scheme 36a) [56]. From the established typology, this transformation represents a formal [4 + 2] cycloaddition with sole activation of the *o*-QM. The vinyl sulfide serves as a masked olefin, enabling indirect access to chromanes otherwise difficult to obtain directly. However, the reaction requires low temperature (−20 °C) and a relatively high catalyst loading (10 mol%), reducing operational simplicity. Atom economy is moderate due to the stoichiometric vinyl sulfide (2 equiv) and subsequent derivatization steps. Thus, from a cycloaddition perspective, this protocol provides a valuable indirect strategy for diversely substituted chiral chromanes.

In a complementary approach, Schneider’s team developed a relay catalytic strategy using **cat 20** for the asymmetric addition of in situ-generated *o*-QMs **140** with β-dicarbonyl compounds **141** (Scheme 36b) [57]. From the established typology, this formal [4 + 2] cycloaddition proceeds via Mn(dbm)_3_/O_2—_mediated aerobic oxidation of 2-alkylphenols to form the *o*-QM, followed by manganese phosphate-catalyzed enantioselective Michael addition, cyclization, and dehydration to afford chiral 4*H*-chromenes **142**. The method uses O_2_ as the terminal oxidant and operates at room temperature. However, it requires a relatively high catalyst loading (10 mol% Mn(dbm)_3_) and is limited to electron-rich phenols. Atom economy is moderate due to the use of excess β-dicarbonyl (1.5 equiv). Thus, from a cycloaddition perspective, this relay catalysis provides an aerobic entry to chiral chromenes, albeit with substrate limitations.

Subsequently, in 2020, Shao’s group reported an asymmetric formal [4 + 2] cycloaddition of in situ-generated *o*-QMs **143** with *ortho*-alkenyl naphthols/phenols **144** using chiral phosphoric acid catalysts (Scheme 36c) [58]. From the established typology, this transformation represents a formal [4 + 2] cycloaddition. Distinct catalyst systems were employed: ***cat 21*** gave *transcis* chromans **145** from 1-enyl-2-naphthols, while **cat 22** gave trans–trans chromans **146** from 2-enyl-1-naphthols. The method affords trisubstituted chromans in excellent yields (up to 99%), enantioselectivities (up to 99% ee), and diastereoselectivities (d.r. >20:1). However, the reaction requires substrate-specific catalysts, limiting operational simplicity. Atom economy is moderate due to the use of stoichiometric 4 Å molecular sieves. Thus, from a cycloaddition perspective, this work provides a stereodivergent entry to chiral chromans with high stereocontrol, albeit with catalyst-dependent substrate specificity.

**Scheme 37:**
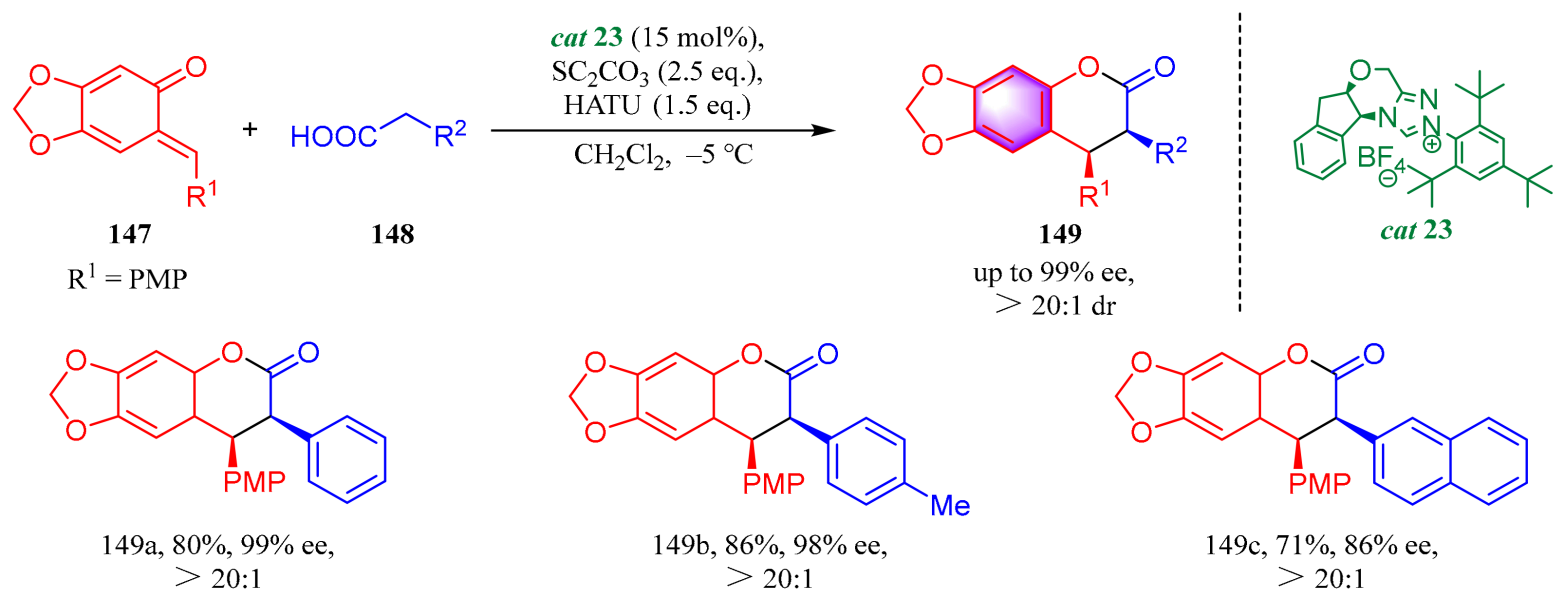
Exploration of the asymmetric [4 + 2] cyclization reaction of *o*-QMs catalyzed by NHC based on in situ activation of saturated carboxylic acids.

In 2017, Yao and co-workers reported an N-heterocyclic carbene (**cat 23**)-catalyzed asymmetric [4 + 2] cyclization of saturated carboxylic acids with in situ-generated *o*-QMs **147/148**, affording chiral dihydrocoumarins **149** (Scheme 37) [59]. From the established typology, this transformation represents a [4 + 2] cyclization. The reaction uses HATU as the condensation reagent and Cs_2_CO_3_ as the base, with **cat 23** (15 mol%) at −5 °C. The method gives products in moderate to good yields (60–86%) with excellent enantioselectivities (up to 99% ee) and good diastereoselectivities (up to >20:1 dr). However, the protocol requires a stoichiometric amount of HATU (1.5 equiv) and a relatively high catalyst loading, reducing atom economy. Additionally, the substrate scope is limited to arylacetic acids; alkyl-substituted butyric acid gave only moderate yield (65%). Thus, from a cyclization perspective, this NHC-catalyzed strategy provides an enantioselective entry to dihydrocoumarins, albeit with moderate atom economy and substrate restrictions.

**Scheme 38:**
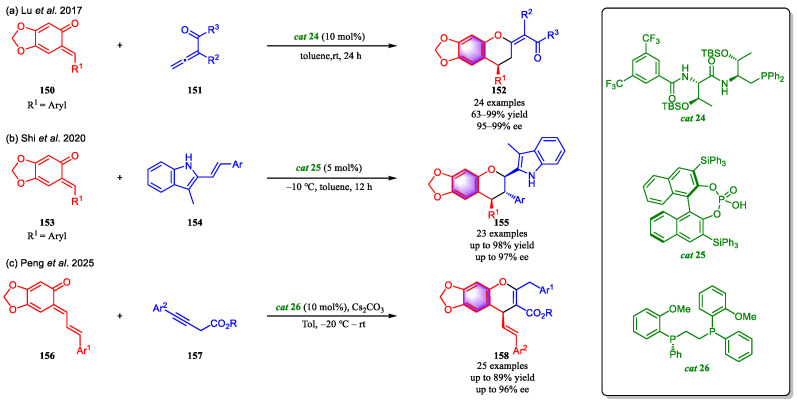
Expansion of C2 synthons in *o*-QMs asymmetric [4 + 2] cyclization reactions: from dienes, alkenyl indoles to alkyne ester precursors. (**a**) Lu et al. 2017 [60]. (**b**) Shi et al. 2020 [61]. (**c**) Peng et al. 2025 [62].

In 2017, Lu et al. reported the first phosphine-catalyzed asymmetric [4 + 2] cyclization of in situ-generated *o*-QMs **150** with allene ketones **151**, affording chiral chromanes **152** (Scheme 38a) [60]. From the established typology, this transformation represents a [4 + 2] cyclization. The reaction uses a dipeptide phosphine catalyst ***cat 24*** (10 mol%) and 4 Å molecular sieves at room temperature, giving products in high yields (63–99%) and excellent enantioselectivities (95–99% ee). The substrate scope tolerates various aryl and alkyl substituents on the allene ketones. However, the protocol requires the use of stoichiometric molecular sieves, and the catalyst loading (10 mol%) is relatively high, which reduces atom economy. Thus, from a cyclization perspective, this work represents the first application of *o*-QMs in phosphine-catalyzed annulation, providing an efficient entry to chiral chromanes, albeit with moderate atom economy.

In 2020, the Shi research group developed a chiral phosphoric acid-catalyzed formal [4 + 2] cycloaddition of *o*-QMs **153** with 3-methyl-2-vinylindole **154**, affording indolyl-substituted chiral 1,3-dioxolochromans **155** (Scheme 38b) [61]. From the established typology, this transformation represents a formal [4 + 2] cycloaddition. The reaction uses **cat 25** (5 mol%) at room temperature, delivering products in moderate to high yields (up to 98% yield) with excellent enantioselectivities (up to 97% ee). Control experiments indicated that hydrogen-bonding interactions between the catalyst and the N-H moiety of the indole are essential for stereocontrol. However, the protocol requires stoichiometric 4 Å molecular sieves, reducing atom economy. Thus, from a cycloaddition perspective, this method expands the scaffold diversity of chiral chromans.

In 2025, Peng and co-workers reported a chiral tertiary phosphine-catalyzed formal [4 + 2] cycloaddition of *o*-QMs **156** with γ-aryl-substituted alkynoates **157**, affording chiral 4*H*-chromenes **158** (Scheme 38c) [62]. From the established typology, this transformation represents a formal [4 + 2] cycloaddition via in situ isomerization of the alkynoate to a γ-substituted allenoate. The reaction uses **cat 26** (10 mol%) and Cs_2_CO_3_ (1.2 equiv) at −20 °C, giving products in good yields (up to 89% yield) and excellent enantioselectivities (up to 96% ee). The substrate scope tolerates various aryl substituents on both components. However, the protocol requires a stoichiometric base and a relatively high catalyst loading, reducing atom economy. Thus, from a cycloaddition perspective, this work provides the first example of using alkynoates as allenoate precursors in asymmetric [4 + 2] cycloadditions with *o*-QMs, albeit with moderate atom economy.

**Scheme 39:**
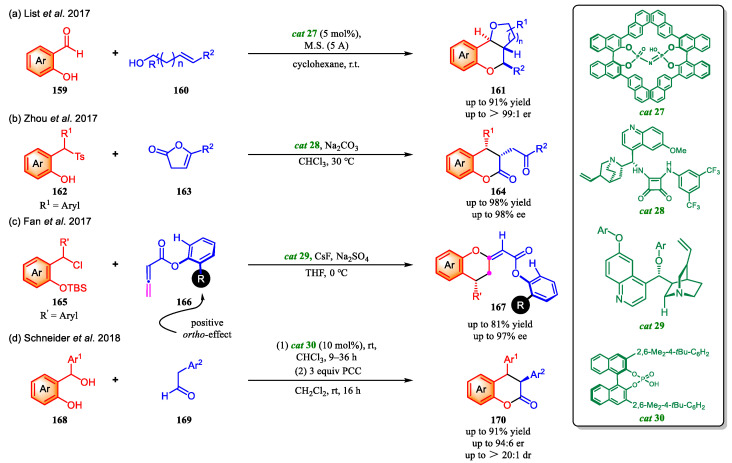
From enols to aldehydes: expanding the nucleophile scope in *o*-QM asymmetric formal [4 + 2] cycloaddition reactions. (**a**) List et al. 2017 [63]. (**b**) Zhou et al. 2017 [64]. (**c**) Fan et al. 2017 [65]. (**d**) Schneider et al. 2018 [66].

In 2017, List reported the first catalytic asymmetric intramolecular formal [4 + 2] cycloaddition of in situ-generated *o*-QMs using a confined chiral imidodiphosphoric acid catalyst (**cat 27**) (Scheme 39a) [63]. Salicylaldehydes and dienyl alcohols gave furano- and pyranochromanes in 75–91% yield, >20:1 dr, and up to 99:1 er. The method features broad scope and kinetic resolution. However, it requires 5 Å molecular sieves, 5 mol% **cat 27**, and up to 48 h; 6-substituted salicylaldehydes are not tolerated. Atom economy is moderate. Thus, from a cycloaddition perspective, this work provides an entry to chromane-based scaffolds, albeit with moderate atom economy.

In the same year, Zhou and co-workers reported an asymmetric α-addition/transesterification of *o*-QM precursors **162** with deconjugated butenolides **163** using a chiral squaramide catalyst **cat 28** (Scheme 39b) [64]. From the established typology, this transformation represents a formal [4 + 2] cycloaddition via an α-addition/transesterification cascade. The reaction proceeds in CHCl_3_ at 30 °C with 10 mol% catalyst, affording 3,4-dihydrocoumarins **164** bearing two contiguous stereocenters in high yields (up to 97%) with excellent enantioselectivities (up to 99% ee) and good diastereoselectivities (up to 9.2:1 dr). DFT calculations suggested that the unusual α-regioselectivity arises from distortion energy differences between transition states. However, the substrate scope is limited: phenols lacking electron-donating groups on the aromatic ring do not react, and 2-(phenyl(tosyl)methyl)phenol gave no product. The reaction requires a stoichiometric base (K_3_PO_4_) and a relatively high catalyst loading (10 mol%), reducing atom economy. Thus, from a cycloaddition perspective, this work provides the first example of asymmetric α-addition of deconjugated butenolides to *o*-QMs, albeit with moderate atom economy and substrate restrictions.

Also in 2017, Fan reported an asymmetric formal [4 + 2] cycloaddition of *o*-QM precursors with allenic esters using a cinchona alkaloid catalyst (**cat 29**) [65]. From the established typology, this formal cycloaddition gave chromanes in 35–81% yield and up to 97% ee. *Ortho*-substituents on the allenic ester improved reactivity/selectivity. However, stoichiometric CsF/Na_2_SO_4_ and high catalyst loading (10 mol%) reduce atom economy. Substrate scope is limited to *ortho*-substituted aryl groups. Thus, this work expands *o*-QMs chemistry in amine catalysis, albeit with moderate atom economy.

In 2018, Schneider reported a chiral phosphoric acid-catalyzed formal [4 + 2] cycloaddition of in situ-generated *o*-QMs with aryl acetaldehydes, giving cis-3,4-diarylchromanols (up to 91% yield, up to 94:6 er, up to >20:1 dr) [66]. The chromanols are versatile intermediates. However, the reaction requires stoichiometric 3 Å molecular sieves and 10 mol% **cat 30**, and it fails with aliphatic or α-branched aldehydes. Thus, this work provides a route to bioactive chroman scaffolds, albeit with limitations in atom economy and aldehyde scope.

**Scheme 40:**
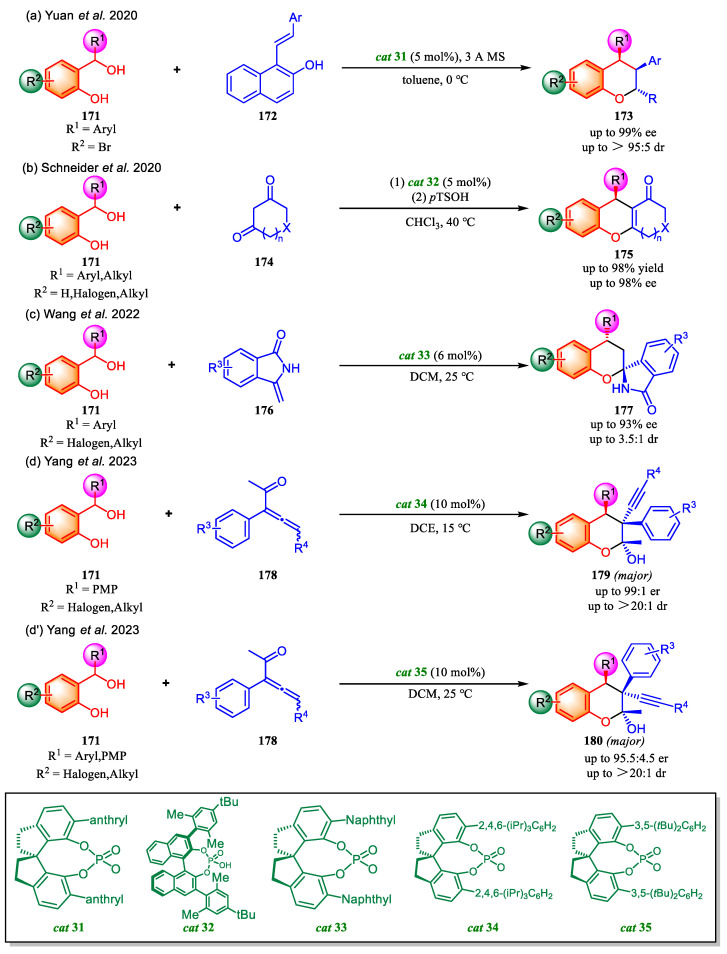
From skeleton expansion to three-dimensional divergence: asymmetric formal [4 + 2] cycloaddition reactions involving *o*-QMs. (**a**) Yuan et al. 2020 [67]. (**b**) Schneider et al. 2020 [68]. (**c**) Wang et al. 2022 [69]. (**d**) Yang et al. 2023 [70]. (**d’**) Yang et al. 2023 [70].

In 2020, Yuan et al. reported an asymmetric formal [4 + 2] cycloaddition of 1-((2-aryl)vinyl)-2-naphthol **172** with *o*-QMs, catalyzed by a chiral phosphoric acid (**cat 31**) (Scheme 40a) [67]. This transformation proceeds via a dearomatization/re-aromatization sequence, enabling the construction of multisubstituted chiral chromane derivatives **190** with excellent enantioselectivities and high diastereoselectivities.

In the same year, Schneider et al. reported a detailed study on a Brønsted acid-catalyzed enantioselective synthesis of 4*H*-chromenes and 1*H*-xanthen-1-ones **175** via the in situ generation of hydrogen-bonded *o*-QMs from *o*-hydroxybenzyl alcohols **171** (Scheme 40b) [68]. Under the catalysis of chiral phosphoric acid (**cat 32**), various β-dicarbonyl compounds **191** undergo a highly enantioselective formal [4 + 2] cycloaddition. Detailed mechanistic investigations including online NMR, ESI-MS, kinetic studies, and DFT calculations indicate that *o*-QM formation is the rate-limiting step, and that the subsequent cycloaddition proceeds via a concerted yet highly asynchronous pathway.

In 2022, Wang et al. further expanded the scope of nucleophiles by reporting an asymmetric formal [4 + 2] cycloaddition of 3-methylene isoindolinone **176** with *o*-QM precursors **171** (generated in situ from *o*-hydroxybenzyl alcohols), catalyzed by chiral phosphoric acid **cat 33** in DCM at room temperature for 20–48 h (Scheme 40c) [69]. This work enables the construction of a spirocyclic *N*-isoindolinone framework, affording a series of spirocyclic *N,O*-heterocycles **177** bearing tetrasubstituted spirocarbon centers in yields of 30–66% with up to 93% ee. Notably, this strategy transforms relatively planar starting materials into more three-dimensional spirocyclic architectures.

Building on the dual concepts of structural diversification and mechanistic advancement, in 2023, Yang et al. reported a diastereodivergent asymmetric formal [4 + 2] cycloaddition of γ-silyl-substituted allenones **178** with in situ-generated *o*-QMs from precursors **171**, catalyzed by chiral phosphoric acids (10 mol%) in DCM at 25 °C for 12–36 h (Scheme 40d,d′) [70]. By switching between catalysts **cat 34** and **cat 35**, complementary C3-epimeric chromanes were obtained with high diastereoselectivities (up to >20:1 dr) and enantioselectivities (up to 99:1 er), in up to 93% yield. Mechanistic experiments (^1^H NMR monitoring and control reactions) revealed the reversibility of the cycloaddition and the conversion of kinetically favored products into thermodynamically more stable diastereomers, demonstrating catalyst-controlled stereodivergence in *o*-QM- based cycloadditions.

**Scheme 41:**
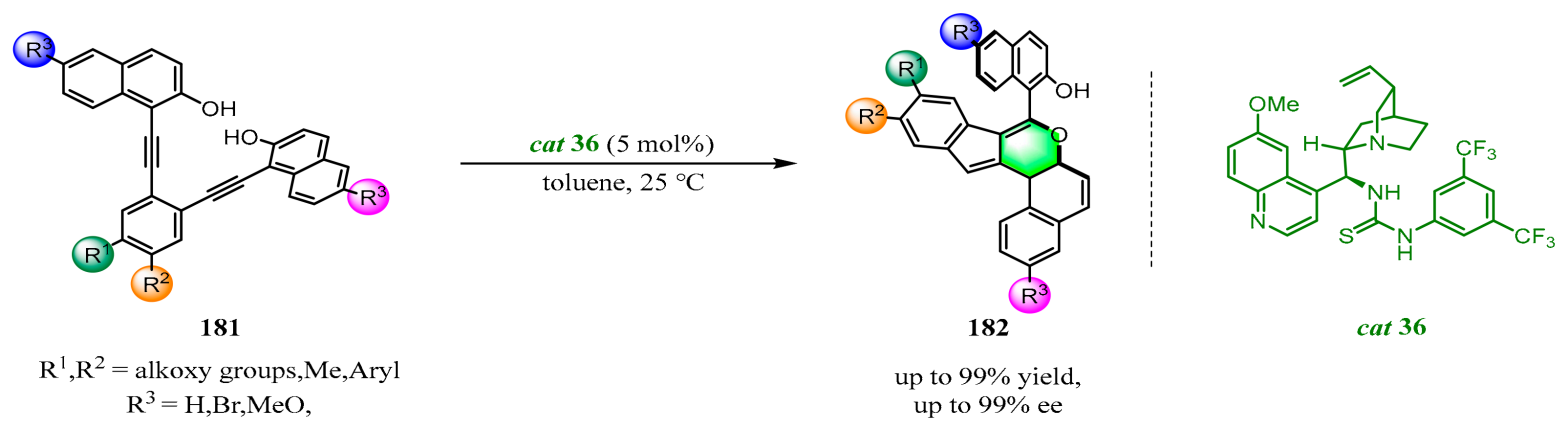
Asymmetric catalytic construction of axially chiral oxygen-heterolinked aryl skeletons: intramolecular formal [4 + 2] cycloaddition reactions involving vinylidene o-Quinone methides.

In 2018, Yan and co-workers reported a chiral thiourea-catalyzed intramolecular formal [4 + 2] cycloaddition of vinylidene *o*-quinone methides (VQMs) generated in situ from 2-ethynylphenol derivatives **181**, affording axially chiral aryl-naphthopyrans **182** (Scheme 41) [71]. From the established typology, this transformation represents an intramolecular formal [4 + 2] cycloaddition. The reaction proceeds in toluene at room temperature with 5 mol% **cat 36**, giving products in excellent yields (up to 99%) and enantioselectivities (up to 99% ee). Mechanistic studies, including control experiments with substrates lacking free hydroxyl groups, indicated that a hydrogen-bonding network between the two OH groups of the substrate and the thiourea/tertiary amine functionalities of the catalyst is essential for both reactivity and stereocontrol. However, the requirement for two free hydroxyl groups limits the substrate scope, and the catalyst loading (5–10 mol%) is relatively high, which moderately reduces atom economy. Thus, from a cycloaddition perspective, this work provides an efficient entry to axially chiral *O*-heterobiaryls, albeit with moderate atom economy.

**Scheme 42:**
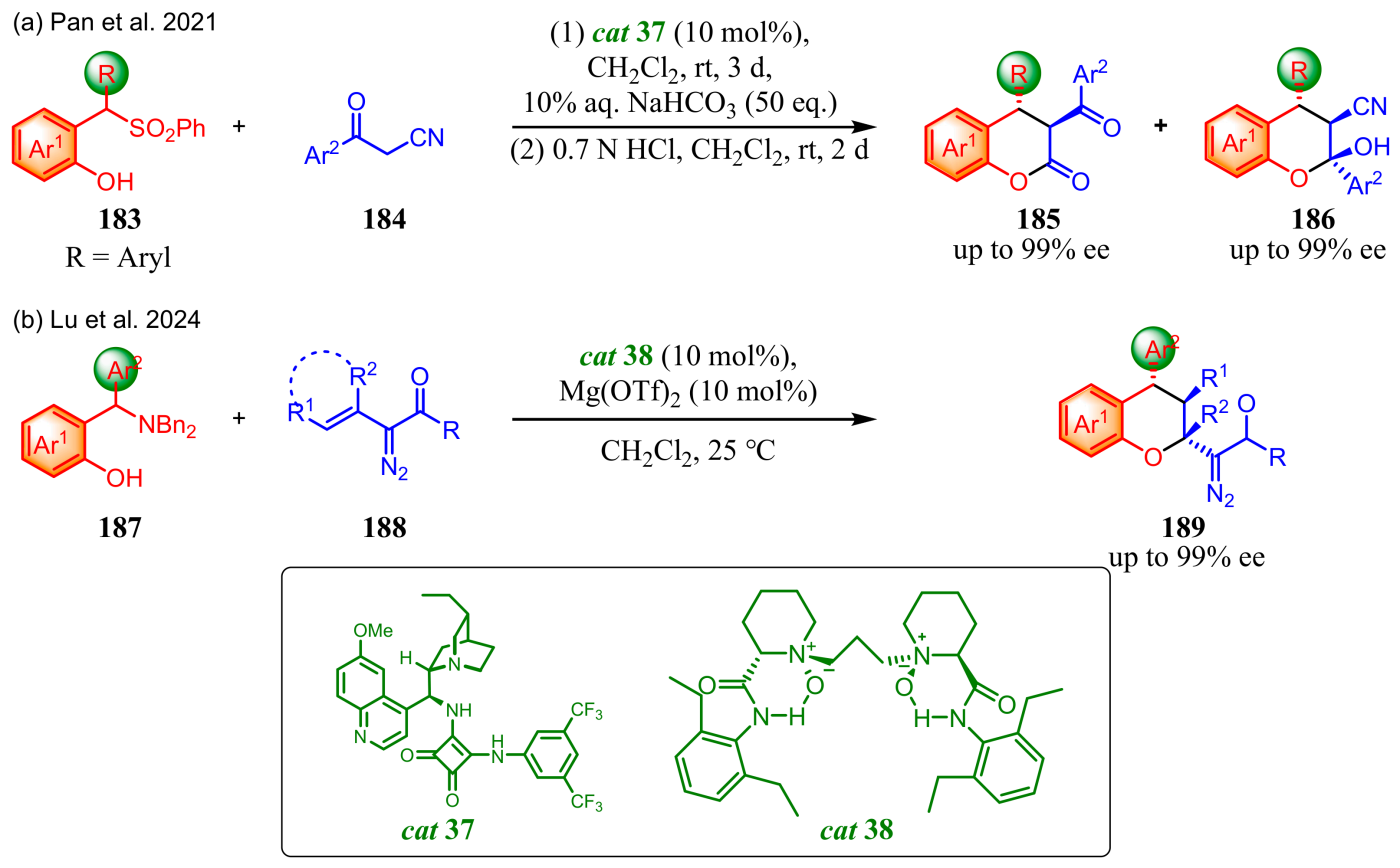
From cyano ketones to diazo compounds: expansion of nucleophiles in *o*-QM asymmetric annulation reactions. (**a**) Pan et al. 2021 [72]. (**b**) Liu et al. 2024 [73].

In 2021, the Pan group disclosed an asymmetric formal [4 + 2] cycloaddition of α-cyanoketones **184** with in situ-generated *o*-QMs catalyzed by **cat 37**, giving 3,4-disubstituted dihydrocoumarins **185** and tetrasubstituted chromans **186** with excellent enantioselectivities (up to 99% ee) (Scheme 42a) [72]. This was the first use of aromatic α-cyanoketones in asymmetric *o*-QM chemistry. Limitations include lower enantioselectivity for a heteroaromatic substrate (2-furoyl, 78% ee) and for an aliphatic ethyl-substituted substrate (70% ee), exclusive formation of the chroman product from a benzylic aliphatic substrate, significantly eroded selectivity with a 3-chloro substituent on the phenol ring (58% ee), and the need for a two-step acidic workup to suppress chroman formation.

In 2024, Liu et al. reported an asymmetric cyclization of vinyl diazo compounds **188** with *o*-QMs catalyzed by a chiral magnesium(II)–N,N′-dioxide complex (**cat 38**) (Scheme 42b) [73]. This strategy represents the first asymmetric transformation of vinyl diazo substrates via a non-carbene pathway, enabling the synthesis of chiral diazo-containing chromane derivatives **189** with excellent enantioselectivities (up to 99% ee) and full diastereocontrol (>20:1 dr). Control experiments and DFT calculations support an ionic stepwise mechanism in which the nucleophilicity of the vinyl group is enhanced by the adjacent diazo moiety. However, several limitations were noted: alkyl-substituted *o*-QM precursors failed to react; electron-deficient naphthol substrates gave lower enantioselectivities; only highly electron-rich phenols were compatible; methyl-substituted and cyclic vinyl diazoacetates afforded products in limited yields with poor enantioselectivities; and the catalyst loading was relatively high (10 mol%).

**Scheme 43:**
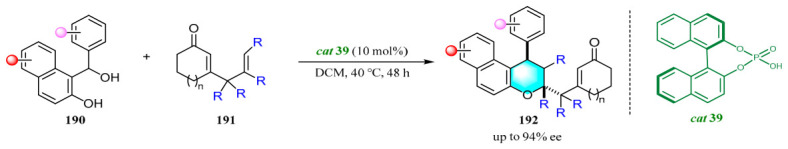
Brønsted acid-catalyzed remote bisvinylogous formal [4 + 2] cycloaddition for naphthopyran synthesis.

In 2026, Singh and co-workers reported a Brønsted acid (**cat 39**)-catalyzed formal [4 + 2] cycloaddition featuring a remote bisvinylogous initiation (Scheme 43) [74]. Using trienol intermediates derived from β-allyl cycloalkenones, the ε-carbon of the dienophile selectively attacks in situ-generated *o*-QMs, enabling efficient construction of naphthalene-pyran fused scaffolds **192** with high diastereoselectivity (up to >20:1 dr). In the asymmetric variant, a *BINOL*-derived chiral phosphoric acid afforded one product in 94% ee when a sterically demanding 4-tert-butyl substituent was present. However, some limitations were observed: alkyl-substituted *o*-QM precursors gave only trace amounts of product; replacing the γ-hydrogen atoms of the cycloalkenone with methyl groups did not lead to the desired product; acyclic bisvinylogous precursors proved unsuitable; and most chiral products were obtained with moderate enantioselectivities (≤53% ee), with the high 94% ee achieved only for a single substrate bearing a bulky group.

## 4. [4 + 3] Cyclization Reactions: Access to Seven-Membered Rings

The construction of seven-membered rings remains a significant challenge in organic synthesis due to both entropic and enthalpic constraints. In this context, [4 + 3] cyclization reactions involving *o*-QMs have emerged as powerful and efficient strategies for the assembly of these valuable molecular scaffolds.

### 4.1. Acid-Catalyzed or Base-Promoted [4 + 3] Cyclization

**Scheme 44:**
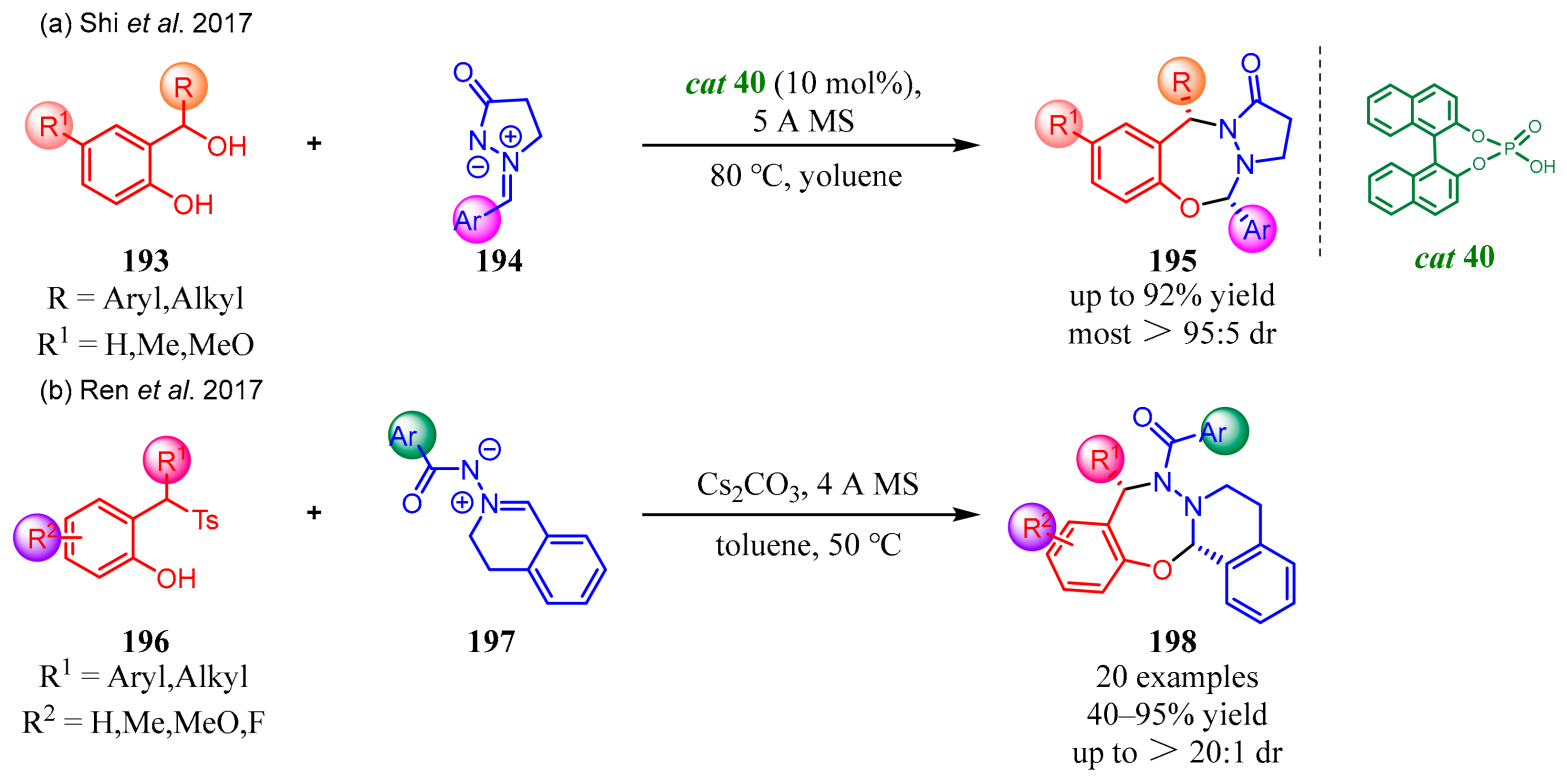
Complementary strategies of acid catalysis and base mediation: formal [4 + 3] cycloaddition of *o*-QMs for the construction of seven-membered oxygen-containing heterocycles. (**a**) Shi et al. 2017 [75]. (**b**) Ren et al. 2017 [76].

In 2017, Shi and co-workers reported a Brønsted acid-catalyzed formal [4 + 3] cycloaddition of *ortho*-hydroxybenzyl alcohols **193** with *N,N’*-cyclic azomethine imines **194** (Scheme 44a) [75]. Using camphorsulfonic acid as the **cat 40**, the reaction afforded benzo-oxadiazepine derivatives **195** in good yields (up to 92%) and excellent diastereoselectivities (most >95:5 dr). This work represents the first formal [4 + 3] cycloaddition employing *ortho*-hydroxybenzyl alcohols as *o*-QM precursors. However, the enantioselective variant was not developed, and a strongly electron-withdrawing group (CN) required elevated temperature (100 °C), leading to lower diastereoselectivity (75:25 dr).

During the same year, Ren and co-workers reported a base-mediated diastereoselective formal [4 + 3] cycloaddition of 2-(1-tosylalkyl)phenols **196** with *C,N*-cyclic azomethine imines **197** (Scheme 44b) [76]. Under Cs_2_CO_3_, the in situ-generated *o*-QMs reacts with the imine to construct 1,3,4-oxadiazepine derivatives **198** in moderate to good yields (up to 95%) and excellent diastereoselectivities (up to >20:1 dr). This base-promoted protocol complements the acid-catalyzed strategy of Shi. However, several limitations were observed: substrates with an ethyl group at the benzylic position gave a dramatically lower yield; electron-withdrawing substituents on the phenol ring required a higher temperature; and the enantioselective variant was not successful in preliminary attempts.

**Scheme 45:**
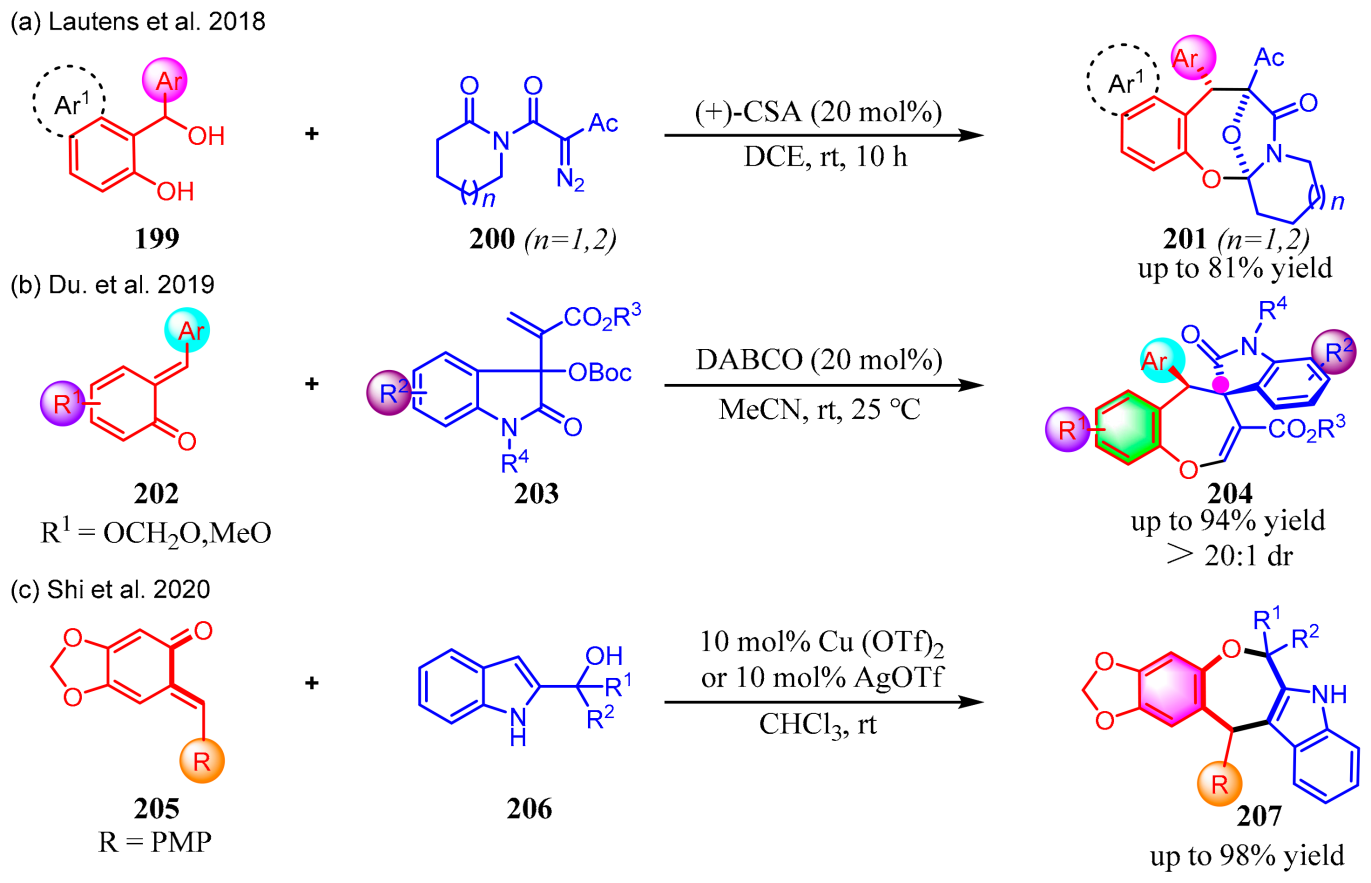
Diversification of three-carbon partners in *o*-QMs [4 + 3] cyclization: from diazo compounds to indolylmethanols. (**a**) Lautens et al. 2018 [77]. (**b**) Du. et al. 2019 [78]. (**c**) Shi et al. 2020 [79].

Lautens group described the formal [4 + 3] cycloaddition reaction of diazoimide **200** with *o*-QMs catalyzed by camphorsulfonic acid in 2018 (Scheme 45a) [77]. This strategy simultaneously and in situ generated two active intermediates, isomünchnone and *o*-QMs, through acid catalysis, and constructed a series of oxygen-bridged oxazocine derivatives **201** with good yield, avoiding the use of metal catalysts. However, the reaction required a high catalyst loading (20 mol% camphorsulfonic acid) and showed limited tolerance for substituents on the diazoimide partner.

In 2019, Du et al. reported a diastereoselective formal [4 + 3] cycloaddition reaction of stable *o*-QMs **202** with MBH carbonates **203** catalyzed by DABCO (Scheme 45b) [78]. This strategy activates **203** with a Lewis base to form an ammonium ylide intermediate, and a series of benzo[b]oxepine derivatives containing a spirooxindole skeleton **204** were constructed in high yields and with good diastereoselectivities (dr >20:1). Nevertheless, the enantioselectivity was not reported, and the reaction was only demonstrated on stable *o*-QMs, not in situ-generated ones.

In 2020, the Shi group reported a Lewis acid-catalyzed C3-nucleophilic [4 + 3] cyclization of stable *o*-QMs **205** with 2-indolylmethanols **206** (Scheme 45c) [79]. This strategy, for the first time, used metal Lewis acids as catalysts to explore the C3-nucleophilic reactivity of **206**, and constructed a series of indole-fused seven-membered heterocyclic derivatives **207** in high yields (up to 98%) with regiospecificity. Nevertheless, the reaction required metal catalysts (Cu(OTf)_2_ or AgOTf), and N-protected 2-indolylmethanols were not tolerated under the standard conditions.

**Scheme 46:**
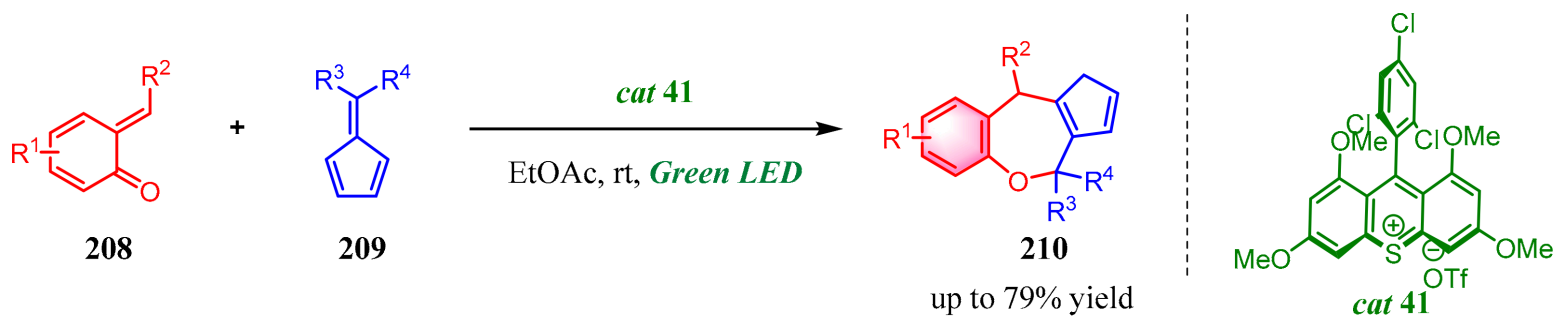
Photocatalytic *o*-QMs [4 + 3] cyclization reactions.

**Scheme 47:**
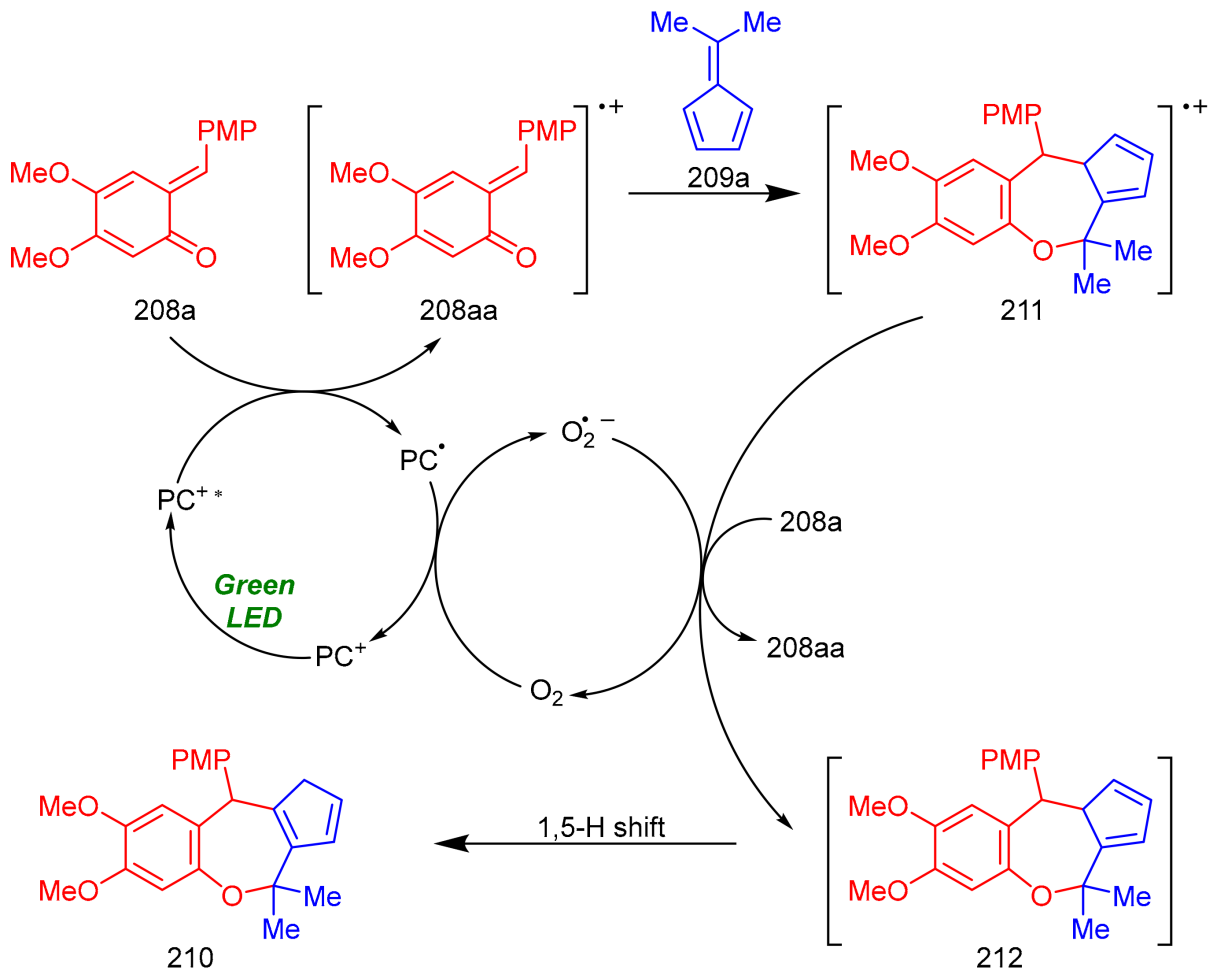
Proposed mechanism for the visible-light-driven formal [4 + 3] cycloaddition of *o*-QMs with fulvenes via radical cation intermediates.

In 2022, Hoshino et al. reported what the authors originally described as a formal [6 + 4] cycloaddition of *o*-QMs **208** with pentafulvenes **209** under photoredox **cat 41** (Scheme 46) [80]. However, from a mechanistic perspective, this transformation can be re-categorized as a formal [4 + 3] cycloaddition (see the catalytic cycle in Scheme 47). Control experiments and DFT calculations support a radical cation pathway initiated by single-electron transfer from the *o*-QM to the excited photocatalyst, followed by a 1,5-Hydrogen shift to afford the thermodynamically favored benzo[b]cyclopenta[e]oxepines **210**.

**Scheme 48:**
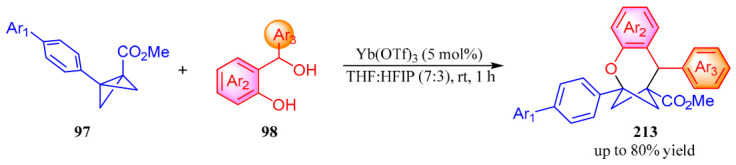
Lewis acid-catalyzed formal [4 + 3] cycloaddition of in situ-generated *o*-QMs and BCBs for sp^3^-rich oxabicyclic frameworks.

In 2026, Saha et al. reported a formal [4 + 3] cycloaddition of in situ-generated *o*-QMs (from precursors **98**) with bicyclo[1.1.0]butanes **97**, catalyzed by Yb(OTf)_3_ in a 7:3 THF/HFIP mixed solvent system (Scheme 48) [44]. The reaction afforded oxabicyclo[4.1.1]octane derivatives **213** in moderate to good yields (up to 80%) with high regioselectivity, providing an efficient route to sp^3^-rich bridged frameworks. However, limitations were noted: the catalyst loading (5 mol%) was not further optimized for lower levels; and the substrate scope was primarily demonstrated on aromatic-substituted precursors, with limited exploration of aliphatic variants.

### 4.2. Asymmetric Catalysis in [4 + 3] Cyclization

**Scheme 49:**
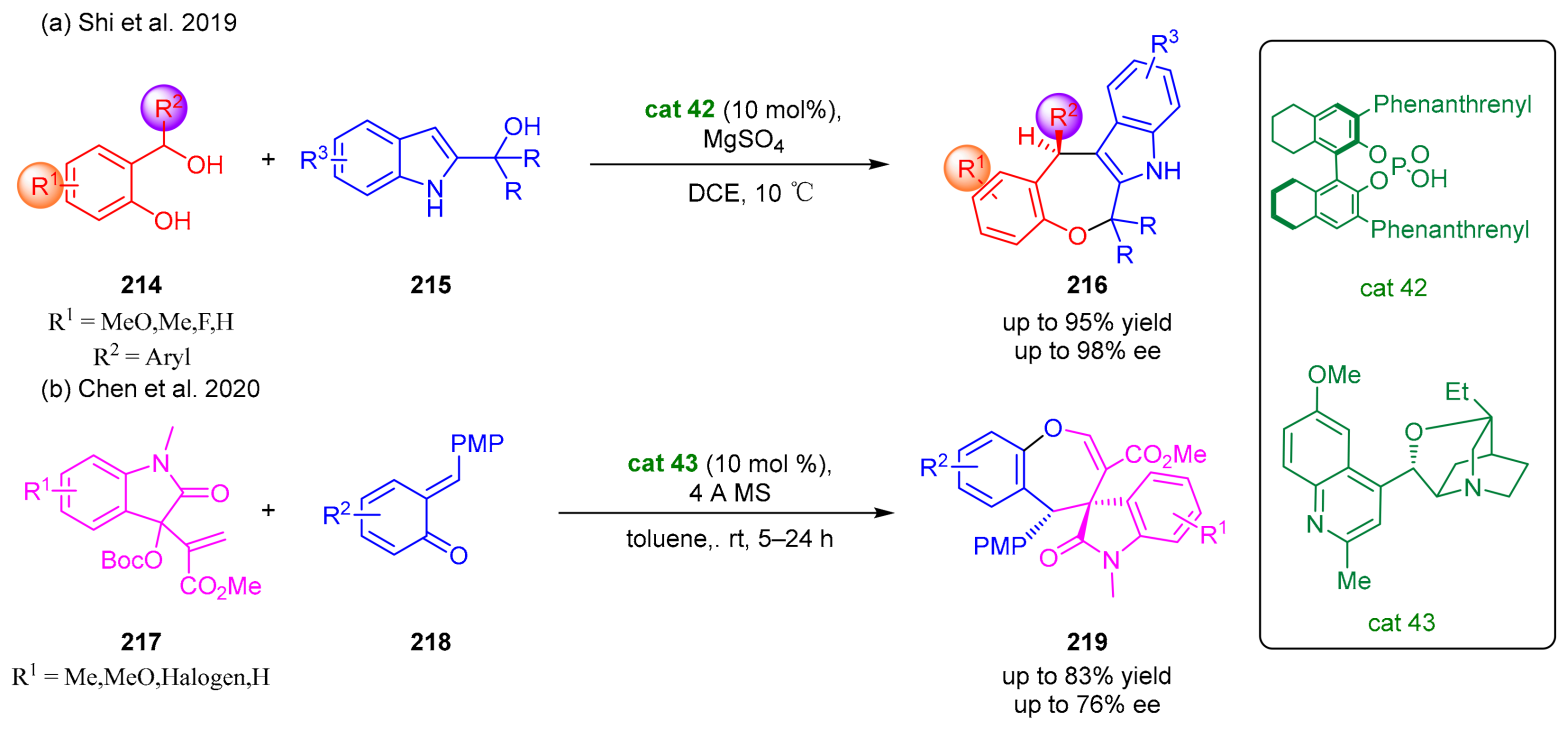
Chiral organocatalyst-catalyzed asymmetric formal [4 + 3] annulations of *o*-QMs. (**a**) Shi et al. 2019 [81]. (**b**) Chen et al. 2020 [30].

In 2019, Shi and co-workers reported an asymmetric [4 + 3] cyclization of *o*-hydroxybenzyl alcohols **214** with 2-indolylmethanols **215**, catalyzed by a chiral phosphoric acid (**cat 42**) (Scheme 49a) [81]. This transformation proceeds via a dual hydrogen-bond activation mode, affording indole-fused seven-membered oxygen heterocycles **216** in high yields (up to 95%) with excellent enantioselectivities (up to 98% ee). This work represents the first asymmetric [4 + 3] cyclization employing *o*-hydroxybenzyl alcohols as *o*-QM precursors, as well as the first asymmetric [4 + 3] cyclization of 2-indolylmethanols. However, some limitations were observed: *N*-protected 2-indolylmethanols were not tolerated; the reaction required a relatively high catalyst loading and extended reaction time; and disubstituted o-hydroxybenzyl alcohols bearing aliphatic groups gave only moderate yields.

In 2020, the Chen research group reported a γ-regioselective formal [4 + 3] cycloaddition of Morita–Baylis–Hillman (MBH) carbonates **217** with *o*-QMs **218**, catalyzed by a chiral tertiary amine (**cat 43**) (Scheme 49b) [30]. This method provides access to chiral oxepane spirooxindole frameworks **219** in good yields with moderate enantioselectivities (up to 76% ee). Notably, simple recrystallization was found to significantly enhance the enantiomeric purity of the cycloadducts (up to 98% ee). However, some limitations were observed: the reaction required a relatively high catalyst loading (20 mol%) and low temperature (−20 °C); the substrate scope was limited (only a few *o*-QM variants were tested); and the enantioselectivities before recrystallization remained moderate for most products.

**Scheme 50:**
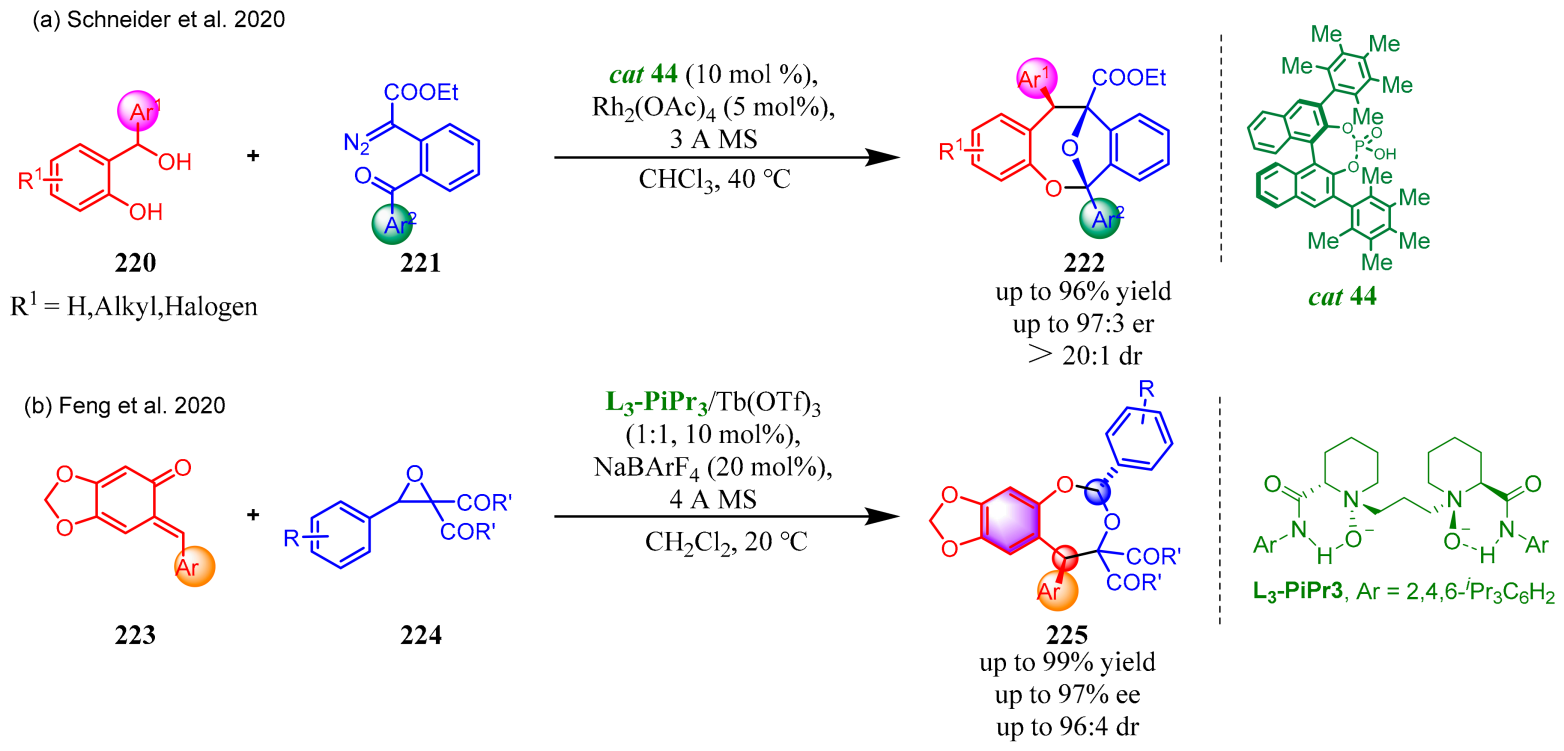
Asymmetric formal [4 + 3] cycloaddition reaction of *o*-QMs mediated by carbonyl-ylide. (**a**) Schneider et al. 2020 [82]. (**b**) Feng et al. 2020 [83].

In 2020, Schneider and co-workers reported an asymmetric formal [4 + 3] cycloaddition of substrate **220** with diazo esters **221**, enabled by cooperative rhodium and chiral phosphoric acid catalysis (Scheme 50a) [82]. In this transformation, a rhodium-catalyzed process generates a carbonyl ylide intermediate, while the chiral phosphoric acid (**cat 44**) activates the *o*-QMs, facilitating a stereoselective formal [4 + 3] cycloaddition. This strategy affords oxygen-bridged dibenzoxacine derivatives in good yields with excellent diastereoselectivities and enantioselectivities, representing the first example of cooperative rhodium/chiral phosphoric acid catalysis for formal [4 + 3] cycloaddition of *o*-QMs with carbonyl ylides. However, some limitations were observed: the reaction required slow addition of the diazoester to avoid side reactions; *ortho*-substituted aryl groups on the diazoester led to diminished diastereo- and enantioselectivity; and electron-poor benzhydryl alcohols gave only moderate yields.

In 2021, Feng et al. reported an asymmetric formal [4 + 3] cycloaddition of *o*-QMs **223** with oxiranes **224**, catalyzed by a chiral *N,N′*-dioxide/Tb(III) complex (Scheme 50b) [83]. In this approach, carbonyl ylides are generated via metal-catalyzed epoxide ring opening, which subsequently undergo formal [4 + 3] cycloaddition with *o*-QMs to furnish hydrodioxepine derivatives **225** in excellent yields (up to 99% yield) and good to excellent enantioselectivities (up to 97% ee). Notably, this work represents the first metal-catalyzed formal [4 + 3] cycloaddition between *o*-QMs and epoxides. Furthermore, the influence of the chiral catalytic environment on stereoselectivity was elucidated through steric analysis of the catalyst framework. However, some limitations were observed: the reaction required the addition of a sodium salt additive (NaBAF_4_) to achieve optimal enantioselectivity; the substrate scope of epoxides was primarily limited to those bearing electron-withdrawing ester groups; and only a limited set of *o*-QM variants were investigated.

## 5. Higher-Order Cycloadditions: [4 + 4] and Beyond

The extension of *o*-quinone methide (*o*-QM) cycloaddition chemistry to higher-order ring systems represents a frontier in this field, providing access to medium-sized rings that are commonly found in natural products yet remain challenging to construct using conventional synthetic approaches.

### [4 + 4] Cyclization

**Scheme 51:**
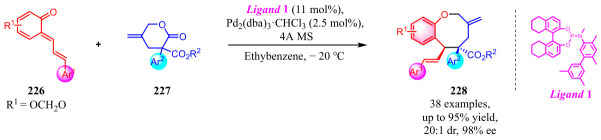
Asymmetric high-order formal cycloaddition reactions of *o*-QMs catalyzed by palladium.

In 2022, Li and co-workers reported an asymmetric formal [4 + 4] cycloaddition of *o*-QMs **226** with γ-methylidene-δ-valerolactones **227**, catalyzed by a palladium system using Pd_2_(dba)_3_ and a chiral phosphoramidite **ligand 1** (Scheme 51) [84]. This strategy represents the first palladium-catalyzed asymmetric formal [4 + 4] cycloaddition of *o*-QMs to construct eight-membered benzo[b]oxocine derivatives **228** bearing adjacent all-carbon quaternary and tertiary stereocenters, affording products in good yields with excellent diastereo- and enantioselectivities. DFT calculations support that the stereoselectivity arises from the preferential addition of a 1,4-dipolar intermediate to the Si face of the *o*-QM. However, some limitations were observed: the reaction required low temperature and the addition of 4 Å molecular sieves; a specific ratio of catalyst to ligand was necessary; the substrate scope of the lactone component was primarily limited to aryl-substituted variants; and the yield decreased slightly upon scale-up.

**Scheme 52:**
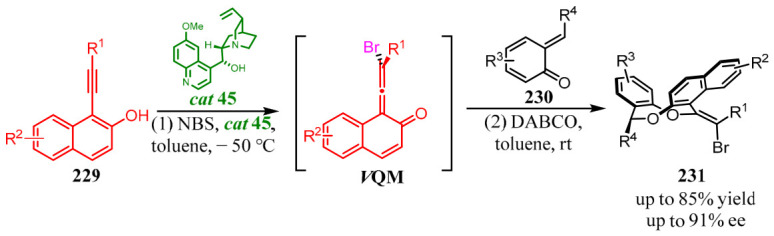
Formal cross-[4 + 4] cycloaddition reactions involving *o*-QMs.

In 2025, Yan and co-workers reported an asymmetric formal cross-[4 + 4] cycloaddition of VQMs **229** with *o*-QMs **230**, catalyzed by cinchona alkaloids (**cat 45**) (Scheme 52) [85]. This transformation proceeds via a one-pot, two-step protocol, enabling the efficient construction of eight-membered oxygen-containing heterocycles **231** possessing inherent chirality in good yields with good to excellent enantioselectivities. Notably, this work represents the first asymmetric formal cross-[4 + 4] cycloaddition of *o*-QMs to access inherently chiral eight-membered *O*-heterocycles. DFT calculations elucidated the origins of chemo- and diastereoselectivity and confirmed that the resulting saddle-shaped heterocycles exhibit high conformational stability. However, some limitations were observed: the reaction required a low temperature (−50 °C) for the first step and a specific two-step procedure; the catalyst loading was relatively high (10 mol%); and although the substrate scope was broad, certain substituents (e.g., *ortho*-substituted arenes) were not explored.

**Scheme 53:**
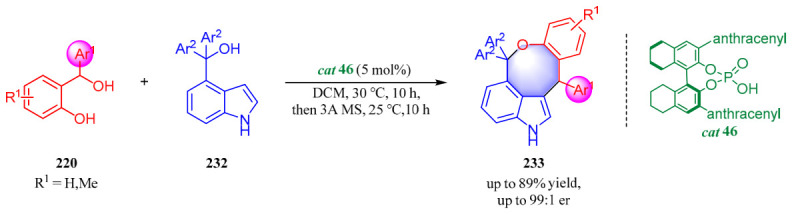
CPA-catalyzed enantioselective [4 + 4] annulation of o-hydroxybenzyl alcohols.

In 2025, Shi and co-workers reported the first enantioselective [4 + 4] annulation between o-hydroxybenzyl alcohols **220** and 4-indolylmethanols 232, catalyzed by a chiral phosphoric acid (**cat 46**) (Scheme 53) [86]. In this transformation, *o*-QM intermediates are generated in situ from **220** and undergo sequential 1,4-addition to the C3-nucleophilic site of 4-indolylmethanols, followed by intramolecular cyclization. This cascade process enables the efficient construction of structurally diverse indole-based eight-membered heterocycles **233** with high enantioselectivities. This strategy fills a key gap in asymmetric formal [4 + 4] annulation chemistry involving *o*-QMs and represents the first example employing 4-indolylmethanol derivatives. However, some limitations were observed: the reaction required a two-step addition of molecular sieves to achieve satisfactory yields; N-methylated 4-indolylmethanols gave poor enantioselectivity; and the substrate scope of 4-indolylmethanols was limited to diaryl-substituted variants, with monoaryl substrates being less reactive.

## 6. Summary and Outlook

Over the past decade, *o*-quinone methide (*o*-QM) cycloaddition chemistry has evolved into a versatile and powerful platform for the construction of diverse carbocyclic and heterocyclic frameworks. Significant advances include the expansion of cycloaddition modes beyond the classical [4 + 2] paradigm to encompass [4 + 1], [4 + 3], [4 + 4] processes, the development of highly enantioselective catalytic systems, and increasingly sophisticated mechanistic insights that enable rational reaction design. Collectively, these methodologies have found broad application in the synthesis of natural products, pharmaceuticals, and functional materials, effectively bridging the gap between fundamental research and practical utility.

Looking ahead, the field is well positioned for continuous innovation through the development of new catalytic systems, as well as the incorporation of biocatalytic and photochemical activation strategies. The integration of emerging technologies such as flow chemistry and machine learning offers additional opportunities to enhance reaction efficiency, predictability, and scalability. However, key challenges still remain, including the development of underexplored asymmetric higher-order cycloadditions, expansion of compatible reaction partners, and improvement of scalability for industrial applications. Given its unique capacity to access structurally complex and medicinally relevant scaffolds, o-QM cycloaddition chemistry is expected to remain a dynamic and impactful area of research, driving future advances in synthetic methodology and complex molecule synthesis.

## Data Availability

The original contributions presented in this study are included in the article. Further inquiries can be directed to the corresponding authors.
